# Synthesis of novel cyclosiloxane monomers containing push–pull moieties and their anionic ring opening polymerization†

**DOI:** 10.1039/c8ra00707a

**Published:** 2018-02-16

**Authors:** Elena Perju, Eduardo Cuervo-Reyes, Sergiu Shova, Dorina M. Opris

**Affiliations:** Swiss Federal Laboratories for Materials Science and Technology Empa, Laboratory for Functional Polymers Ueberlandstr. 129 CH-8600 Dübendorf Switzerland Dorina.opris@empa.ch; “Petru Poni” Institute of Macromolecular Chemistry of Romanian Academy Aleea Gr. Chica Voda, 41A 700487 Iasi Romania; Swiss Federal Laboratories for Materials Science and Technology Empa, Laboratory for Energy Conversion Ueberlandstr. 129 CH-8600 Dübendorf Switzerland

## Abstract

The synthesis of three novel tetracyclosiloxane monomers modified either with a nitroaniline (NA) or with a Disperse Red 1 (DR1) push–pull group and their ring opening polymerization reaction in the presence of tetramethylammonium hydroxide are presented. The prepared monomers and polymers were characterized by different spectral methods and gel permeation chromatography. For the crystalline monomers, the structures were further proven by single crystal X-ray diffraction. Dynamic scanning calorimetry shows that the polymers that carry NA groups have a glass transition temperature (*T*_g_) well below room temperature (RT), while the one that carries DR1 groups melts at 55 °C. The transition temperatures have a strong effect on permittivity as indicated by broadband impedance spectroscopy measurements conducted at different temperatures and frequencies. The polymers modified with NA groups have a high permittivity (maximum value of 17.3) at RT, suggesting the polar groups to be mobile and orientation polarization to be effective. However, the polar groups of the polymer modified with DR1 are frozen and thus cannot contribute to the permittivity *via* orientation polarization. Consequently, the permittivity is only 8.8 at RT, but increases to 22 above the melting temperature, where the dipoles are mobile. Because of the high dielectric permittivity and rather low *T*_g_, the polymers modified with NA are attractive as active dielectric materials in actuators, capacitors, and stretchable electronics, whereas the polymer modified with DR1 may be of interest in nonlinear optical devices.

## Abstract 2

The synthesis of polysiloxanes modified with a nitroaniline or with a Disperse Red 1 push–pull group and their dielectric properties are reported.

## Graphical abstract

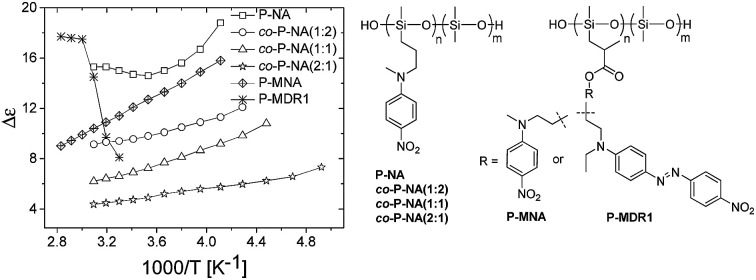

## Introduction

Push–pull molecules such as nitroaniline (NA) and disperse red 1 (DR1) have a large dipole moment and are often used as active components in nonlinear optic (NLO) polymer devices to increase the NLO coefficient^1–4^ as well as in electromechanical transducers to increase the polarizability and thus the dielectric permittivity (*ε*′).^5^ In nonlinear optical devices, the active polymers are non-centrosymmetric and have the push–pull moieties aligned in the same direction and in a frozen-in state. Such orientation is achieved *via* the poling in which the initially randomly oriented dipoles of a polymer are poled in a strong electric field above the glass transition temperature (*T*_g_).^6^ By cooling the sample below *T*_g_ while maintaining the electric field, the oriented structure is frozen and the poled polymer shows NLO properties. For this application, polymers with a high *T*_g_ and high density of push–pull moieties are required.

For the second application, however, these moieties have to be mobile to be able to contribute to the dielectric permittivity *via* orientation polarization. Orientation polarization is effective for low *T*_g_ polymers which have permanent dipoles incorporated. When a polar, low *T*_g_ polymer is exposed to an electric field, the dipoles align in a certain time along the electric field direction. Depending on the size of the dipole used, they may contribute to the permittivity beyond a certain frequency.^7–15^

Push–pull groups can be introduced on polysiloxane chain using a post-polymerization modification reaction. This has been achieved starting from either azide or hydrosilyl containing polysiloxanes.^16^ The azides were subsequently used to introduce nitrobenzene groups *via* aza-click chemistry,^17^ while the hydrosilyl groups were used in hydrosilylation reactions with push–pull compounds that carry a reactive double bond. Using this strategy, Yang and Wnek introduced silyl ketene acetal pendant groups which were subsequently reacted with 4-nitrobenzenesulphenyl chloride.^18,19^ Kussmaul *et al.* grafted *N*-allyl-*N*-methylaniline to the Si–H groups of a poly(methylhydro-*co*-dimethyl)siloxane in an *in situ* process optimized to produce elastomers, but only a rather small amount of push–pull dipoles was incorporated as reflected by the rather low permittivity of about *ε*′ = 6 achieved for their materials.^20–22^ Racles *et al.* introduced DR1 groups on a polysiloxane chain using a condensation reaction.^23^ Unfortunately, in all cases the amount of polar groups incorporated was rather low and therefore the increase in permittivity was only moderate. To the best of our knowledge, no cyclosiloxane monomers exist that carry push–pull dipoles. Such monomers would provide access to the corresponding polysiloxanes combining high content of polarizable groups with main chain flexibility. The current work therefore describes how some of these monomers can be synthesized and converted into polymers. We further present the glass transition temperatures of these polymers and their dielectric permittivity at different temperatures and frequencies. Depending on their particular transition temperatures, these novel polymers may find application as dielectric in mechanical actuators or as active component in non-linear optical devices.

## Results and discussion

**Scheme 1:**
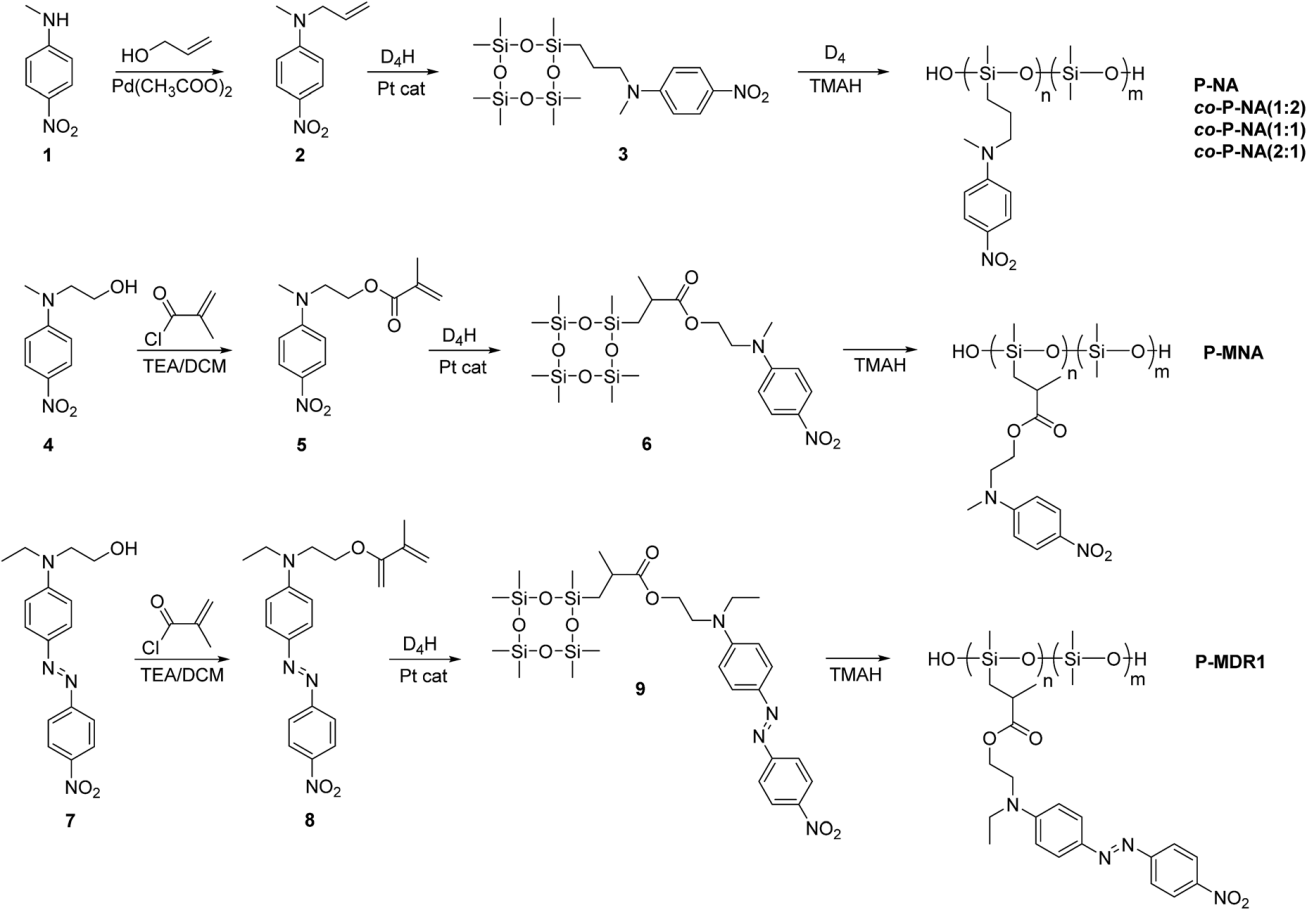
Synthesis of cyclosiloxanes containing nitroaniline and Disperse Red 1 groups and their ring opening polymerization.

**Fig. 1:**
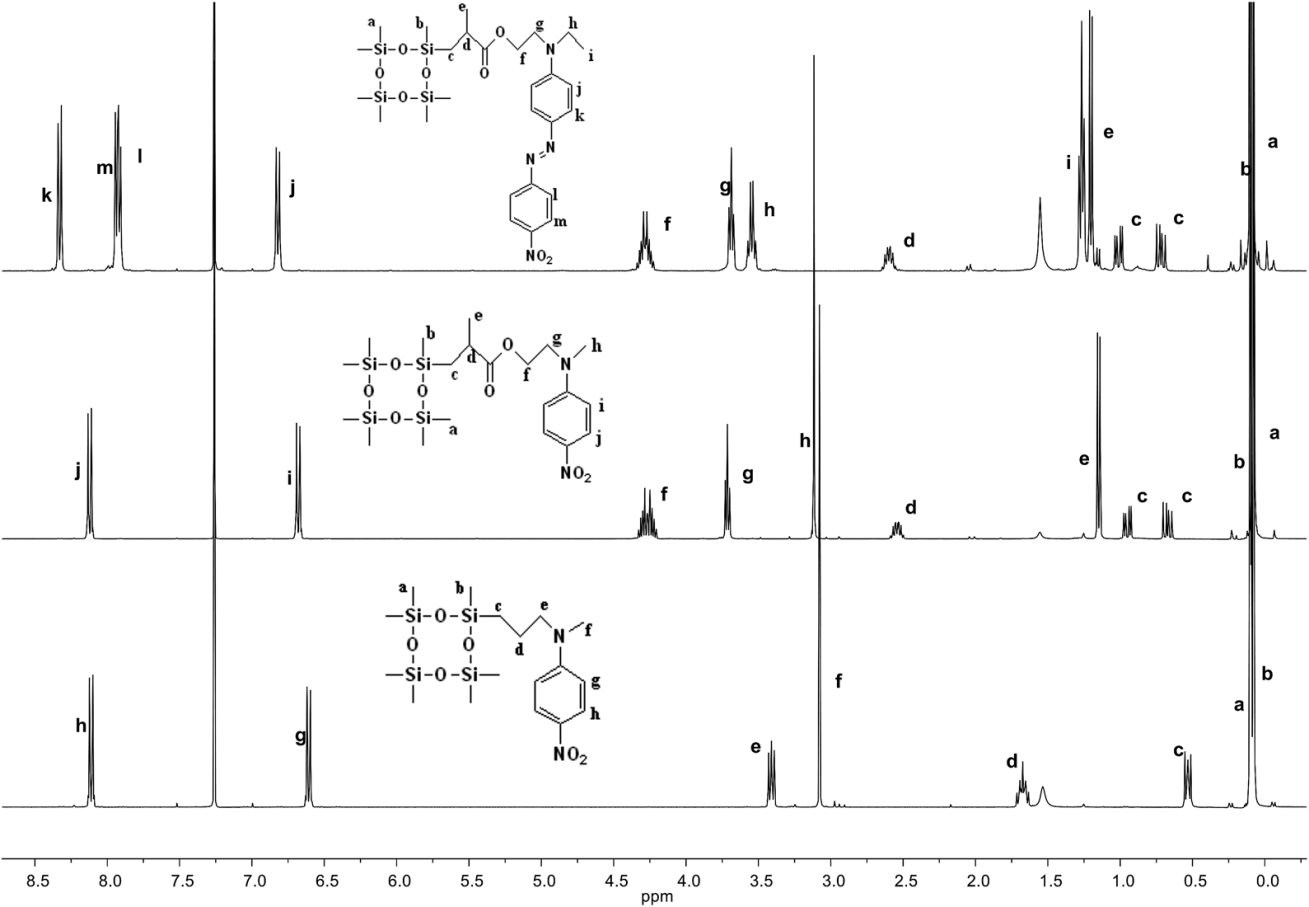
^1^H NMR spectra of cyclosiloxane monomers 3 (bottom), 6 (middle), and 9 (top) that carry a push–pull group.

The synthetic strategy to the cyclosiloxanes monomers modified either with NA or DR1 push–pull moieties is illustrated in Scheme 1. They were prepared *via* a hydrosilylation reaction of heptamethylcyclotetrasiloxane (D_4_H) with NA or DR1 modified with allyl or methacryl groups. For this, *N*-methyl-*p*-nitroaniline 1 was reacted with allyl alcohol to form 2 which carries a reactive allyl group. This group was subsequently used in a hydrosilylation reaction with D_4_H to give the tetracyclosiloxane monomer 3 that carries a nitroaniline group. Fig. S1† shows the ^1^H NMR spectra of the two starting compounds D_4_H and 2 and of the product 3. The disappearance of the hydrosilyl group at 4.7 ppm of D_4_H and of the allyl group between 5 to 6 ppm and the appearance of new signals in the aliphatic part of 3 are clear indicators of the successful hydrosilylation. The other two monomers, 6 and 9, were prepared *via* a hydrosilylation reaction of D_4_H either with NA or DR1 moieties 5 or 8 that were modified with a methacryl group. While monomers 3 and 6 formed in rather high yields, monomer 9 modified with DR1 was obtained only in a moderate yield of 30%. The monomers were purified by column chromatography over silica gel and they were isolated as viscous oils. They were pure compounds as proven by their elemental analysis and ^1^H NMR spectra (Fig. 1). Compound 3 and 9 slowly solidified at room temperature (RT).

**Fig. 2:**
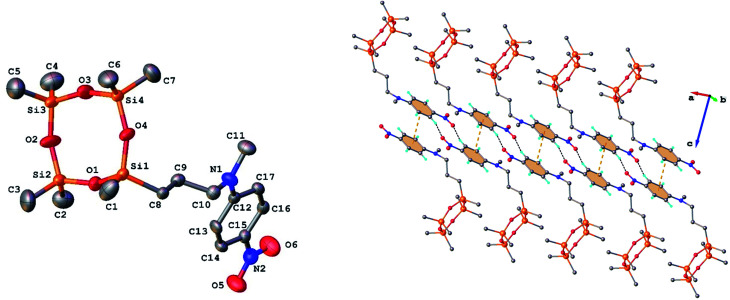
Left: Molecular structure of compound 3 in the crystal (ORTEP) with atom labeling scheme. H-atoms omitted for clarity. Right: Packing in the crystal shows a one-dimensional supramolecular architecture. Centroid-to-centroid contacts (3.724(5) Å) are drawn in dashed orange and H-bonds in dashed black lines. H-bond parameters: C16–H⋯O6 [C16–H 0.930 Å, H⋯O6 2.505 Å, C16⋯O6′(−2−*x*, 2−*y*, 1−*z*) 3.375(6) Å, ∠C16HO6 155.8°].

**Fig. 3:**
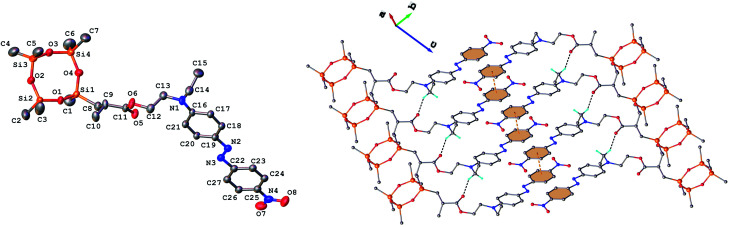
Left: Molecular structure of compound 9 (ORTEP) with atom labeling scheme. H-atoms omitted for clarity. Only the major component of two disordered models is shown. Right: Packing in the crystal shows a one-dimensional supramolecular architecture. Centroid-to-centroid contacts (3.735(6) Å) are drawn in dashed orange and H-bonds in dashed black lines. H-bond parameters: C15–H⋯O5 [C15–H 0.960 Å, H⋯O5 2.392 Å, C15⋯O5′(−1 +*x*, *y*, *z*) 3.359(6) Å, ∠C15HO5 148.5°] (right).

Single crystals of 3 and 9 were grown from ethanol and their solid state structure was established by X-ray diffraction. Both monomers have a molecular crystal structure with *P*1̄ space group of triclinic system. The asymmetric part of the unit cell comprises one discrete molecule as shown in Fig. 2 and 3. The bond lengths and angles are summarized in Table 1S (ESI†). Analysis of the packing diagrams indicates a close similarity of compounds 3 and 9. In the crystal, the asymmetric units are linked through weak C–H⋯O hydrogen bonds and π–π stacking interactions. The main crystal structure motif can be characterized as a parallel packing of infinite supramolecular ribbons along *a* axis, as shown in Fig. 2 and 3.

The ring opening polymerization of 3, 6, and 9 as well as the copolymerization of 3 with octamethylcyclosiloxane (D_4_) was performed. Polymerization of cyclosiloxanes can be conducted in cationic as well as in anionic conditions, however due to the presence of the amino groups in the monomers, which can interfere with the cationic initiator, it was decided to conduct anionic ring-opening polymerization.^24^ As initiator tetramethylammonium hydroxide (TMAH) was used, since it allows for a facile purification of the polymers. This also enabled to introduce silanol end-groups which we intended to use further for cross-linking in thin films to elastomers.^25^

All reactions were conducted at 100 °C and monomers 3, 6, and 9 polymerized to highly viscous liquids P-NA, P-MNA, and P-MDR1, respectively. For the copolymerization of 3 different ratios of monomers D_4_ : 3 (*x* : *y*) were used: 2 : 1, 1 : 1, and 1 : 2 (see Table 1) to furnish copolymers *co*-P-NA(*x*:*y*) with different contents of NA. With increasing the amount of 3, an increase in the signals of the aromatic part, characteristic for the NA moiety, was observed. The ^1^H NMR spectra of the polymers were broader as compared to the starting monomer. The molecular weight and dispersity were determined by GPC using tetrahydrofuran solvent and polydimethylsiloxane standards (Table 1). For the copolymers a molar mass increase was noted with increasing content of D_4_ in the copolymerization mixture. The polymerization of 3 afforded polymers of a higher *M*_w_ as compared to the one obtained from 6 and 9. All polymers showed a rather broad PDI, characteristic for polysiloxanes obtained under thermodynamic equilibrium. Slight deviations of the theoretical content of the push–pull moieties and the one found by ^1^H NMR was observed (Table 1).

**Table 1:** Amount of reagents used for the synthesis of (*co*)polymers, their molecular weights and dispersions as well as the theoretical and calculated ratio of siloxy units that carry a push–pull group to dimethylsiloxy one

Sample	3, 6 or 9		D_4_		*M*_w_	*M*_n_	PDI	Si(polar)CH_3_ : Si(CH_3_)_2_^a^	Si(polar)CH_3_ : Si(CH_3_)_2_^b^
	[g]	[mmol]	[g]	[mmol]	[Da]	[Da]			
P-NA	2	4.2	—	—	196 200	57 600	3.41	1 : 3	1 : 2.70
*co*-P-NA(1:2)	2	4.2	0.62	2.1	229 700	88 600	2.59	1 : 5	1 : 4.60
*co*-P-NA(1:1)	1	2.1	0.62	2.1	189 500	72 400	2.62	1 : 7	1 : 5.99
*co*-P-NA(2:1)	1	2.1	1.25	4.2	292 200	115 600	2.53	1 : 11	1 : 9.85
P-MNA	5	9.4	—	—	95 960	36 580	2.62	1 : 3	1 : 3.10
P-MDR1	3	4.6	—	—	78 600	22 600	3.48	1 : 3	1 : 2.94

a Theoretical ratio of siloxy units that carry a push–pull group to dimethylsiloxy.

b Calculated one from ^1^H NMR spectra.

Thermogravimetric analysis shows that all polymers are stable up to 300 °C, where they start to decompose (see ESI†). Differential scanning calorimetry was used to find the phase transition of monomers and polymers. Monomer 3 has a *T*_m_ = 112 °C, while the polymerization of 3 allowed formation of P-NA with a *T*_g_ = −33 °C. As expected with increasing the content of dimethylsiloxy units in the copolymers *co*-P-NA, a decrease in the *T*_g_ was observed (Table 2). Monomer 6 has a larger flexible chain between the NA moiety and the tetracyclosiloxane ring as compared to 3. Monomer 6 is therefore liquid at RT (*T*_m_ = −40 °C). The *T*_g_ of P-MNA was however the same as the one of P-NA. Monomer 9 was solid at RT (*T*_m_ = 44.9 °C). Interestingly, while the polymerization of 3, which is solid at RT, allowed formation of a polymer with a rather low *T*_g_, the ring opening polymerization of 9, which is also solid, gave a polymer with a *T*_m_ = 55.4 °C, likely due to stronger dipolar interactions. While this *T*_m_ may be too low for some NLO properties applications, by increasing the density of DR1 dipoles in the polymer it should be possible to further increase the *T*_m_.

**Table 2:** Thermal data of the prepared monomers and polymers^a^

Entry	*T*_o_ [°C]	*T*_max_ [°C]	Residue [%]	*T*_Cr_ [°C]	*T*_m_ [°C]	*T*_g_ [°C]
3	289	345	1.35	43	112	—
6	329	360	10.6	—	—	−40
9	337	358	20.5	−0.3	44.9	—
P-NA	346	359	36.1	—	—	−33
*co*-P-NA(1:2)	348	363	41.0	—	—	−64
*co*-P-NA(1:1)	344	363	31.8	—	—	−70
*co*-P-NA(2:1)	354	374	35.2	—	—	—
P-MNA	352	364	16.9	—	—	−32
P-MDR1	338	351	25.6	18.2	55.4	—

a *T*_o_: initial decomposition temperature; *T*_max1_: maximum weight loss rate; *T*_Cr_: crystallization temperature; *T*_m_: melting temperature; *T*_g_: glass transition temperature.

**Fig. 4:**
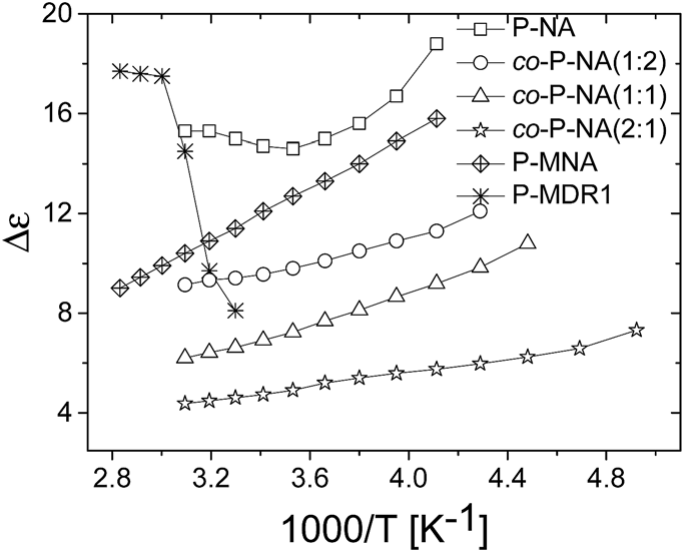
Dielectric enhancement of polysiloxanes modified with NA and DR1 push–pull groups as function of 1/*T*.

To find how the dielectric properties of the prepared polymers are affected by the presence of push–pull groups, dielectric spectroscopy measurements were performed at different temperatures and frequencies. The measured dielectric response contains in all cases a dipolar and a conductivity contribution from mobile ions.^26^ Therefore, we modelled the data using the following expression for the complex permittivity:
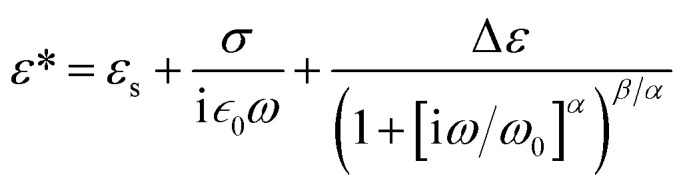


The background permittivity (*ε*_s_), the dipolar enhancement (Δ*ε*), the conductivity (*σ*), the characteristic dipolar activation frequency (*ω*_0_), and the characteristic exponents (*α* and *β*) of the dipolar loss peak were extracted from the data fit at every temperature and their temperature dependence is shown in Fig. 4 and ESI.† To ensure the correctness of the fit, we also extracted the parameters from fits to the electric modulus and verified that both results are consistent. Here the parameters in the temperature range where the fits were conclusive are presented. For instance, the data at temperatures below 30 °C could not be analyzed as the signal is dominated by the nearly constant loss from caged ions. At the highest temperatures, the loss peak falls out of the frequency range and therefore neither the characteristic frequency nor the characteristic exponents can be precisely determined.

**Fig. 5:**
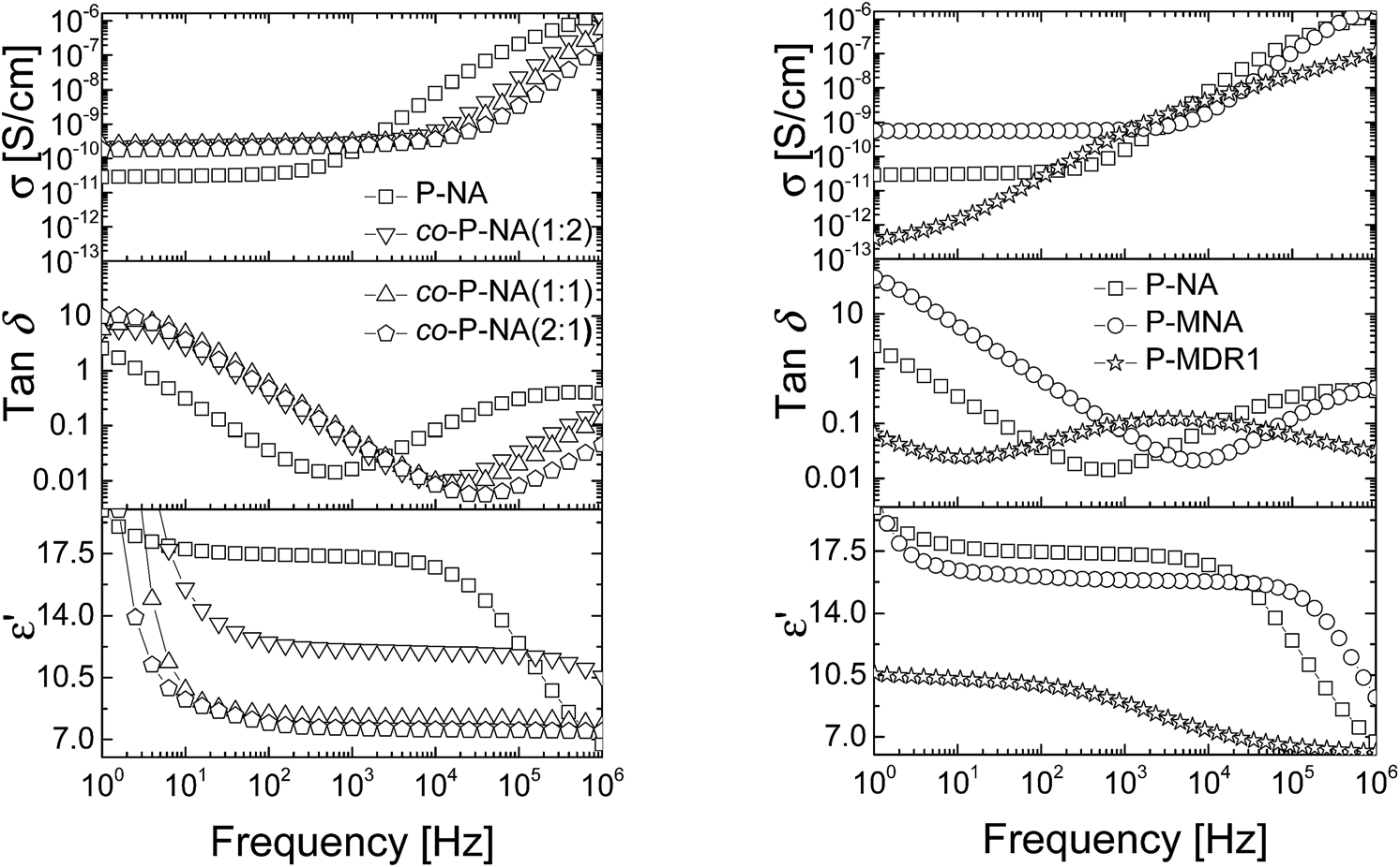
Dielectric permittivity (bottom), dielectric loss (middle) and conductivity (top) of the P-NA and *co*-P-NA(*x*:*y*) (left) and of P-NA, P-MNA, and P-MDR1 (right) as a function of frequency at RT.

The dielectric enhancement shows a consistent increase with the concentration of dipoles and a nearly (1/*T*) dependence on the temperature, with the exception of the P-NA and P-MDR1 which have the highest dipoles concentration and/or melting point above RT. The ratios of the enhancements among *co*-P-NA(1:2), *co*-P-NA(1:1), and *co*-P-NA(2:1) follow closely the ratios of the corresponding concentrations. Thus, with increasing the content of NA in (*co*)polymers an increase in the permittivity was observed from 7.5 for *co*-P-NA(2:1), to 12 for *co*-P-NA(1:2), and reached a maximum value of 17.3 for P-NA which had the maximum content of NA (Fig. 5, Table 3).

**Table 3:** The dielectric properties (permittivity, dielectric losses, loss factor, and conductivity) taken at 10^3^ Hz of the prepared polymers at RT

Entry	*ε*′	*ε*′′	tan *δ*	*σ* [S cm^−1^]
P-NA	17.3	0.26	0.016	1.56 × 10^−10^
*co*-P-NA(1:2)	12.1	0.38	0.044	3.00 × 10^−10^
*co*-P-NA(1:1)	8.2	0.67	0.064	2.97 × 10^−10^
*co*-P-NA(2:1)	7.6	0.15	0.055	2.35 × 10^−10^
P-MNA	15.8	0.39	0.058	6.18 × 10^−10^
P-DR1	8.8	0.96	0.108	6.36 × 10^−10^

Polymers P-NA and P-MNA contain the same NA push–pull group, but their push–pull groups are slightly differently connected to the polymer chain. Because of this, the concentration of push–pull moieties in P-MNA is slightly lower as compared to P-NA, which is reflected by a slightly lower value for the dielectric permittivity at high frequencies. For example P-MNA has a *ε*′ = 15.8, whereas P-NA has a *ε*′ = 17.3 (Fig. 5). Although the dipole moment of DR1 is higher as compared to NA and one would expect that the polymer modified with DR1 to have the highest permittivity, this was not the case. The reason behind this is the *T*_m_ = 55.4 °C of this polymer. Therefore, at RT, the push–pull dipoles are frozen and the orientation of the dipoles in an alternative electric field is not possible at this temperature. However, when heated to temperatures above *T*_m_, where the dipoles are mobile and can contribute to orientation polarization, an increase in permittivity was observed to *ε*′ = 21. Thus, the behavior of P-MDR1 is anomalous due to the strong link among the dipoles in the solid state. P-NA shows the influence of the glass transition as the temperature approaches −30 °C. Consistent with this, the characteristic exponents of the relaxation peaks are systematically smaller for P-MDR1 and P-NA than for the other samples. For an ideal system of non-interacting dipoles *α* = *β* = 1. As the correlations between dipoles become stronger, the exponent 0 < *β* < 1 takes smaller values. Values of 0 < *α* < 1 indicate strongly correlated (glassy) dynamics with power-law (slow) relaxation in time.

Both *α* and *β* increase as the temperature is increased (see ESI†), as expected from the loss of correlation when interaction becomes less relevant in comparison with thermal fluctuations.

**Fig. 6:**
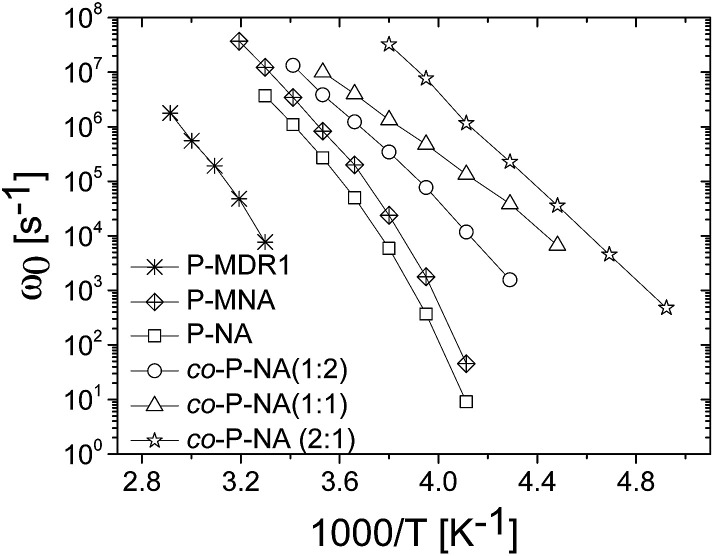
Temperature dependence of the characteristic frequency of the loss peak for all prepared polymers.

The temperature dependence of the characteristic frequency of the loss peak for all samples is shown in Fig. 6. A systematic shifting to lower frequencies with the increase in the dipole–dipole interactions is observed. Characteristic conductivity relaxation times *τ* = (*ε*_s_ + Δ*ε*)*ε*_0_/*σ*, and values of conductivity *versus* temperature as well as separate plots of *τ* and *ω*_0_ for each sample are shown for completeness in ESI.†

Looking at the general trend we conclude that although higher concentrations of dipoles may lead to stronger dielectric enhancement, it may also cause a slower dynamics and loss of dielectric response due to stronger interactions. In this respect, P-MNA was found to be an interesting candidate for applications as actuator as it has a relatively large Δ*ε*, similar to P-MDR1 and P-NA, but also retains larger values for *α* and *β*. This performance could be related to the effective dilution of the dipoles due to the longer chain used for the attachment to the backbone. Although P-MNA has the largest ion conductivity of the set investigated, at high temperatures, it also displays the strongest temperature dependence resulting in ion conductivity similar to those of the P-NA and *co*-P-NA(*x*:*y*) samples at room and lower temperatures.

A direct comparison of the conductivity of the polymers reported here and other high permittivity silicones reported in the literature is not possible, as the conductivity is influenced in a complex way by several factors. The currently highest dielectric permittivity values were reported for silicone based polymers with either nitrile (*ε*′ = 18.2, *T*_g_ = −52.6 °C) or methylsulfone (*ε*′ = 22.7, *T*_g_ = 19.2 °C) groups at every siloxy unit.^5^ The polymers reported here carry polar groups at every fourth siloxy unit. Some of them have a *T*_g_ < RT and therefore a high permittivity at RT. For example, polymers P-NA and P-MNA have *ε*′ = 17.3 and *ε*′ = 15.8, respectively. While these values are slightly lower than the reported ones, the rather low *T*_g_ of P-NA and P-MNA and the possibility of introducing even more polar push–pull groups to the polysiloxane chain offer interesting options. Optimizing these factors will open access to polymers with even higher permittivity or with increased NLO coefficient. Supposed the *T*_g_ of such polymers is below 37 °C, applications in dielectric elastomer actuators for muscle replacement applications can be envisioned.

## Experimental section

### Materials and characterization

*N*-Methyl-4-nitroaniline, allyl alcohol, palladium acetate, triphenyl phosphine (TPP), titanium–isopropoxide, Karstedt's catalyst (platinum(0)-1,3-divinyl-1,1,3,3-tetramethyldisiloxane complex solution in xylene, Pt ∼2%) (Pt cat), methacryloyl chloride, *N*-ethyl-*N*-(2-hydroxyethyl)-4-(4-nitrophenylazo)aniline (Dispersed Red 1), *N*-(2-hydroxyethyl)-*N*-methyl-4-nitroaniline, triethylamine (TEA) and dry benzene were purchased from Sigma-Aldrich. Methanol, heptane, ethyl acetate, dichloromethane, toluene and tetrahydrofuran were purchased from VWR. Tetramethylammonium hydroxide pentahydrate (25% wt in methanol) (TMAH), octamethylcyclotetrasiloxane (D_4_), and heptamethylcyclotetrasiloxane (D_4_H) were purchased from ABCR. Toluene was dried over sodium using benzophenone, and dichloromethane was dried over calcium hydride.

^1^H and ^13^C NMR spectra were recorded on a Bruker Avance III 400 NMR spectrometer in CDCl_3_ using a 5 mm BBO Prodigy™ CryoProbe at 400.18, 100.63 and 79.50 MHz, respectively. Mass spectrometry data were obtained using an Agilent 6520 Series Accurate-Mass Quadrupole Time-of-Flight (Q-TOF) LC/MS. Data were collected and processed using MassHunter Workstation Software Data Acquisition for 6200/6500 Series, version B.01.03. Elemental analysis was determined with LECO TruSpec Micro, LECO RO-478 and LECO CHNS-932.

GPC measurements were taken in THF using an Agilent 1100 Series HPLC (columns: serial coupled PSS SDV 5 μm, 100 Å and PSS SDV 5 μm, 1000 Å, Detector: DAD, 235 nm and 360 nm; refractive index). The calibration was performed with PDMS standards and using toluene as internal standard.

Thermogravimetric analysis (TGA) was conducted with a Perkin Elmer TGA7 at a heating rate of 20 °C min^−1^ under a nitrogen gas flow. Differential scanning calorimetry (DSC) investigations were undertaken on a Pyris Diamond DSC (Perkin Elmer USA) instrument under a nitrogen flow.

The dielectric permittivity was measured using a Novocontrol Alpha-A Frequency Analyzer equipped with a temperature controller. The samples were placed between two golden plated stainless steel electrodes (diameter 20 mm) and the distance between electrodes was adjusted to 100 μm using spacers.

X-ray diffraction measurements for 3 and 9 were carried out with an Oxford-Diffraction XCALIBUR E CCD diffractometer equipped with graphite-monochromated MoKα radiation. Single crystals were positioned at 40 mm from the detector and 365, and 468 frames were measured each for 15, and 50 s over 1° scan width for 3, and 9, respectively. The unit cell determination and data integration were carried out using the CrysAlis package of Oxford Diffraction.^27^ The structures were solved by direct methods using Olex2 and refined by full-matrix least-squares on *F* with SHELXL-97 using an anisotropic model for non-hydrogen atoms.^28,29^ All H atoms were introduced in idealized positions (*d*_CH_ = 0.96 Å) using the riding model with their isotropic displacement parameters fixed at 120% of their riding atom. The positional parameters parts in 9 refined using available tools (PART, DFIX, and SADI) of SHELXL97. The molecular plots were obtained using the Olex2 program. The structure of 9 was refined to a relatively low level solely due to a poor quality of the crystals. CCDC – 1811155 (3) and CCDC – 1811156 (9) contain the supplementary crystallographic data for this contribution.†

#### Synthesis of *N*-allyl-*N*-methyl-4-nitroaniline (2)

The synthesis of *N*-allyl-*N*-methyl-4-nitroaniline was performed according to the literature with slight modifications.^20^

*N*-Methyl-*p*-nitroaniline 1 (1 g, 6.57 mmol), allyl alcohol (0.8 ml, 11.76 mmol), palladium acetate (14.7 mg, 0.066 mmol), triphenyl phosphine (69 mg, 0.263 mmol), and titanium isopropoxide (80 ml, 1.68 mmol) were dissolved in 80 ml dry toluene. The reaction mixture was evacuated, flushed with nitrogen, and heated for 5 h at 115 °C. The water was removed by azeotropic distillation. The solution was filtered, the solvent was removed, and the residue was chromatographed using hexane/ethyl acetate (4 : 1) as eluent. Compound 2 was isolated in a yield of 96% as orange-yellowish oil which crystalized under cooling.

^1^H NMR (400 MHz, CDCl_3_, *δ*): 3.08 (s, 3H, C*H*_3_–N–); 4.02 (m, 2H, –N–C*H*_2_–); 5.11 (d, 1H, –CH=C*H*_2_, *trans*); 5.21 (d, 1H, –CH=C*H*_2_, *cis*); 5.81 (m, 1H, –C*H*=CH_2_); 6.62 (d, 2H, H_Ar_(e)); 8.10 (d, 2H, H_Ar_(f)); ^13^C NMR (100 MHz, CDCl_3_, *δ*): 38.54 (*C*H_3_–N–); 54.90 (–N–*C*H_2_–); 110.61 (–CH=*C*H_2_); 116.99 (–*C*H=CH_2_); 126.12 (C_Ar_(e)); 131.41 (C_Ar_(f)); 137.27 (C_Ar_–NO_2_); 153.50 (C_Ar_–N); Anal. calcd for C_10_H_12_N_2_O_2_: C 62.48, H 6.30, N 14.57, O 16.64; found C 62.47, H 6.27, N 14.74, O 16.53.

#### Synthesis of *N*-(3-(2,4,4,6,6,8,8-heptamethyl-1,3,5,7,2,4,6,8-tetraoxatetrasilocan-2yl)-propyl)-*N*-methyl-4-nitroaniline (3)

A degassed solution of heptamethylcyclosiloxane D_4_H (1 g, 3.53 mmol), 2 (0.82 g, 4.24 mmol), and 1 wt% platinum(0)-1,3-divinyl-1,1,3,3-tetramethyl disiloxane (0.01 g) in 10 ml dry toluene was heated to 110 °C. After all Si–H groups were consumed, the reaction mixture was concentrated and the residue was purified by column chromatography (heptane/ethyl acetate = 4 : 1) to give 3 as yellow crystals with a yield of 94%.

^1^H NMR (400 MHz, CDCl_3_, *δ*): 0.07 (m, 21H, (C*H*_3_)_2_Si–O–; SiC*H*_3_–O–); 0.51 (t, 2H, Si–C*H*_2_–); 1.65 (m, 2H, –CH_2_–C*H*_2_–); 3.06 (s, 3H, C*H*_3_–N–); 3.39 (t, 2H, –N–C*H*_2_–); 6.59 (d, 2H, H_Ar_(g)); 8.09 (d, 2H, H_Ar_(h)); ^13^C NMR (100 MHz, CDCl_3_, *δ*): −0.68 (Si*C*H_3_–O–); 0.70, 0.77 ((*C*H_3_)_2_Si–O–); 14.07 (Si–*C*H_2_–); 20.26 (–CH_2_–*C*H_2_–); 38.70 (*C*H_3_–N–); 55.28 (–N–*C*H_2_–); 110.07 (C_Ar_(g)); 126.68 (C_Ar_(h)); 136.66 (C_Ar_–NO_2_); 153.38 (C_Ar_–N); MS (ESI) *m*/*z* calc. for C_17_H_34_N_2_O_6_Si_4_: 475.815 [M + H]^+^, 497.797 [M + Na]^+^, 513.905 [M + K]^+^; found: 475.081 [M + H]^+^, 497.061 [M + Na]^+^, 513.032 [M + K]^+^; Anal. calcd for C_17_H_34_N_2_O_6_Si_4_: C 42.99, H 7.23, N 5.90; found C 42.97, H 7.18, N 6.02.

##### Crystal data for 3

C_17_H_34_N_2_O_6_Si_4_, Mr = 474.82 g mol^−1^, size 0.40 × 0.30 × 0.03 mm^3^, triclinic, space group *P*1̄, *a* = 6.8337(6) Å, *b* = 8.3901(5) Å, *c* = 22.7542(8) Å, *α* = 86.843(5)°, *β* = 87.831(6)°, *γ* = 84.815(6)°, *V* = 1296.60(16) Å^3^, *Z* = 2, *ρ*_calcd_ = 1.216 g cm^−3^, *μ*(MoKα) = 0.261 mm^−1^, *F*(000) = 508, 9890 reflections in *h*(−7/8), *k*(−10/10), *l*(−28/24), measured in the range 4.88 ≤ *Θ* ≤ 52.74, *T* = 200 K, completeness *Θ*_max_ = 100.0%, 5307 independent reflections, *R*_int_ = 0.0388, 270 parameters, 0 restraints, *R*_1obs_ = 0.0749, w*R*_2obs_ = 0.1282, *R*_1all_ = 0.1194, w*R*_2all_ = 0.1473, GoF = 1.089, largest difference peak and hole: 0.38/−0.29 e A^−3^.

#### Synthesis of P-NA and *co*-P-NA(*x*:*y*)

A solution of tetramethylammonium hydroxide in methanol 25% (TMAH) (1.5 μl) was dried. To the dry TMAH different molar ratios of monomers 3 : D_4_ were added (1 : 0; 1 : 0.5; 1 : 1; 0.5 : 1). The flask was immersed in a preheated oil bath (104 °C) and stirred for 6 h. Then, the temperature was raised to 140 °C for 1 h to decompose the initiator. The reaction mixture was washed several times with methanol and precipitated twice from THF with methanol in order to remove unreacted cycles and oligomers.

##### P-NA

^1^H NMR (400 MHz, CDCl_3_, *δ*): 0.04 (19.32H, (C*H*_3_)_2_Si–O–; SiC*H*_3_–O–); 0.46 (2H, Si–C*H*_2_–); 1.59 (2H, –CH_2_–C*H*_2_–); 3.02 (3H, C*H*_3_–N–); 3.34 (2H, –N–C*H*_2_–); 6.52 (2H, H_Ar_(g)); 8.03 (2H, H_Ar_(h)); ^13^C NMR (100 MHz, CDCl_3_, *δ*): −0.53 (Si*C*H_3_–O–); 1.03, 1.08 ((*C*H_3_)_2_Si–O–); 14.42 (Si–*C*H_2_–); 20.29 (–CH_2_–*C*H_2_–); 38.68 (*C*H_3_–N–); 55.34 (–N–*C*H_2_–); 110.02 (C_Ar_(g)); 126.15 (C_Ar_(h)); 136.57 (C_Ar_–NO_2_); 153.28 (C_Ar_–N); EA calc. for C_17_H_34_N_2_O_6_Si_4_: C 42.99, H 7.23, N 5.90; found C 43.33, H 7.41, N 6.43; GPC: *M*_n_ = 57 618 g mol^−1^; *M*_w_ = 196 220 g mol^−1^; PDI = 3.40.

##### *co*-P-NA(1:2)

^1^H NMR (400 MHz, CDCl_3_, *δ*): 0.04 (30.68H, (C*H*_3_)_2_Si–O–; SiC*H*_3_–O–); 0.48 (2H, Si–C*H*_2_–); 1.61 (2H, –CH_2_–C*H*_2_–); 3.02 (3H, C*H*_3_–N–); 3.37 (2H, –N–C*H*_2_–); 6.54 (2H, H_Ar_(g)); 8.05 (2H, H_Ar_(h)); ^13^C NMR (100 MHz, CDCl_3_, *δ*): −0.51 (Si*C*H_3_–O–); 1.03–1.10 ((*C*H_3_)_2_Si–O–); 14.45 (Si–*C*H_2_–); 20.30 (–CH_2_–*C*H_2_–); 38.75 (*C*H_3_–N–); 55.45 (–N–*C*H_2_–); 110.13 (C_Ar_(g)); 126.20 (C_Ar_(h)); 136.71 (C_Ar_–NO_2_); 153.30 (C_Ar_–N); Anal. calcd for C_21_H_46_N_2_O_8_Si_6_: C 40.47, H 7.46, N 4.50; found C 41.14, H 7.76, N 4.67; GPC: *M*_n_ = 88 596 g mol^−1^; *M*_w_ = 229 680 g mol^−1^; PDI = 2.59.

##### *co*-P-NA(1:1)

^1^H NMR (400 MHz, CDCl_3_, *δ*): 0.05 (38.98H, (C*H*_3_)_2_Si–O–; SiC*H*_3_–O–); 0.47 (t, 2H, Si–C*H*_2_–); 1.62 (2H, –CH_2_–C*H*_2_–); 3.03 (3H, C*H*_3_–N–); 3.36 (2H, –N–C*H*_2_–); 6.54 (2H, H_Ar_(g)); 8.05 (2H, H_Ar_(h)); ^13^C NMR (100 MHz, CDCl_3_, *δ*): −0.52 (Si*C*H_3_–O–); 1.01–1.38 ((*C*H_3_)_2_Si–O–); 14.44 (Si–*C*H_2_–); 20.32 (–CH_2_–*C*H_2_–); 38.67 (*C*H_3_–N–); 55.39 (–N–*C*H_2_–); 110.02 (C_Ar_(g)); 126.19 (C_Ar_(h)); 136.61 (C_Ar_–NO_2_); 153.34 (C_Ar_–N); Anal. calcd for C_25_H_58_N_2_O_20_Si_8_: C 45.15, H 7.59, N 3.63; found C 38.93, H 7.86, N 3.89; GPC: *M*_n_ = 72 393 g mol^−1^; *M*_w_ = 189 510 g mol^−1^; PDI = 2.62.

##### *co*-P-NA(2:1)

^1^H NMR (400 MHz, CDCl_3_, *δ*): 0.04 (61.81H, (C*H*_3_)_2_Si–O–; SiC*H*_3_–O–); 0.49 (2H, Si–C*H*_2_–); 1.63 (2H, –CH_2_–C*H*_2_–); 3.02 (3H, C*H*_3_–N–); 3.37 (2H, –N–C*H*_2_–); 6.56 (2H, H_Ar_(g)); 8.07 (2H, H_Ar_(h)); ^13^C NMR (100 MHz, CDCl_3_, *δ*): −0.51 (Si*C*H_3_–O–); 1.02–1.10 ((*C*H_3_)_2_Si–O–); 14.46 (Si–*C*H_2_–); 20.34 (–CH_2_–*C*H_2_–); 38.70 (*C*H_3_–N–); 55.44 (–N–*C*H_2_–); 110.09 (C_Ar_(g)); 126.23 (C_Ar_(h)); 136.70 (C_Ar_–NO_2_); 153.33 (C_Ar_–N); Anal. calcd for C_16.5_H_41_N_1_O_7_Si_6_: C 37.11, H 7.75, N 2.62; found C 37.67, H 8.12, N 2.76; GPC: *M*_n_ = 115 630 g mol^−1^; *M*_w_ = 292 230 g mol^−1^; PDI = 2.52.

#### 2-(Methyl(4-nitrophenyl)amino)ethyl methacrylate (5)

To an ice-cooled solution of 4 (0.03 mol) and distilled triethylamine (0.05 mol) in dry dichloromethane (27 ml) freshly distilled methacryloyl chloride (0.04 mol) was added slowly under argon atmosphere. The ice bath was removed one hour after methacryloyl chloride was added and the solution was left to react overnight. It was then washed with brine. The organic phase was separated and the solvent was removed. The residue was purified by column chromatography on silica gel using ethyl acetate: heptane (1 : 4) as eluents, to give 5 in over 90% yield.

^1^H NMR (400 MHz, CDCl_3_, *δ*): 1.89 (s, 3H, CH_2_=C(C*H*_3_)–COO–); 3.13 (s, 3H, C*H*_3_–N–); 3.78 (t, 2H, –N–C*H*_2_–); 4.37 (t, 2H, –C*H*_2_–OOC–); 5.57 (s, 1H, C*H*_2_=C(CH_3_)–, *trans*); 6.04 (s, 1H, C*H*_2_=C(CH_3_)–, *cis*); 6.69 (d, 2H, H_Ar_(g)); 8.13 (d, 2H, H_Ar_(h)); ^13^C NMR (100 MHz, CDCl_3_, *δ*): 18.40 (CH_2_=C(*C*H_3_)–COO–); 39.13 (*C*H_3_–N–); 50.95 (–N–*C*H_2_–); 61.50 (–*C*H_2_–OOC–); 110.62 (C_Ar_(i)); 126.3 (C_Ar_(j)); 126.47 (*C*H_2_=C(CH_3_)–); 135.86 (CH_2_=*C*(CH_3_)–); 137.57 (C_Ar_–NO_2_); 153.53(C_Ar_–N); 167.3 (–*C*OO–); Anal. calcd for C_13_H_16_N_2_O_4_: C 59.08, H 6.10, N 10.60; found C 58.81, H 6.14, N 10.64.

#### Synthesis of 2-(methyl(4-nitrophenyl)amino)ethyl-2-(2,4,4,6,6,8,8- heptamethyl-1,3,5,7,2,4,6,8-tetraoxatetrasilocan-2-yl)propanoate (6)

A solution of D_4_H (5 g, 17.67 mmol), 5 (20 mol% excess to the D_4_H) and 1 wt% Karstedt's catalyst in dry toluene (50 ml) was heated to 110 °C overnight under argon. The reaction mixture was concentrated and the residue was purified by column chromatography (heptane : ethyl acetate = 4 : 1) to obtain an organic yellow oil (6) in 85% yield.

^1^H NMR (400 MHz, CDCl_3_, *δ*): 0.08 (m, 21H, (C*H*_3_)_2_Si–O–; SiC*H*_3_–O–); 0.67 (2d, 1H, Si–C*H*_2_–, *trans*); 0.95 (2d, 1H, Si–C*H*_2_–, *cis*); 1.15 (d, 3H, –CH_2_–CH(C*H*_3_)–); 2.55 (m, 3H, –CH_2_–C*H*(CH_3_)–); 3.12 (s, 3H, C*H*_3_–N–); 3.72 (t, 2H, –N–C*H*_2_–); 4.27 (m, 2H, –C*H*_2_–OOC–); 6.68 (d, 2H, H_Ar_(i)); 8.12 (d, 2H, H_Ar_(j)); ^13^C NMR (100 MHz, CDCl_3_, *δ*): 0.21 (Si*C*H_3_–O–); 0.90 ((*C*H_3_)_2_Si–O–); 19.41 (–CH_2_–CH(*C*H_3_)–); 21.61 (Si–*C*H_2_–); 34.81 (–CH_2_–*C*H(CH_3_)–); 39.14 (*C*H_3_–N–); 50.98 (–N–*C*H_2_–); 61.03 (–*C*H_2_–OOC–); 110.60 (C_Ar_(k)); 126.32 (C_Ar_(l)); 137.61 (C_Ar_–NO_2_); 153.53 (C_Ar_–N); 177.51 (–*C*OO–); MS (ESI) *m*/*z* calc. for C_20_H_38_N_2_O_8_Si_4_: 547.878 [M + H]^+^, 569.860 [M + Na]^+^, 585.968 [M + K]^+^; found: 547.088 [M + H]^+^, 569.067 [M + Na]^+^, 585.038 [M + K]^+^; Anal. calcd for C_20_H_38_N_2_O_8_Si_4_: C 43.93, H 7.00, N 5.12; found C 43.71, H 6.94, N 5.00.

#### 2-(Ethyl(4-((4-nitrophenyl)diazenyl)phenyl)amino)ethyl methacrylate (8)

To an ice-cooled solution of *N*-ethyl-*N*-(2-hydroxyethyl)-4-(4-nitrophenylazo)aniline 7 (0.03 mol) and distilled triethylamine (0.05 mol) in dry dichloromethane (27 ml) freshly distilled methacryloyl chloride (0.04 mol) was added slowly under argon atmosphere. The ice bath was removed one hour after methacryloyl chloride was added and the solution was left to react overnight. It was then washed with brine. The organic phase was separated and the solvent was removed. The residue was purified by column chromatography on silica gel using ethyl acetate : heptane (1 : 4) as eluents, to give 8 in over 90% yield.

^1^H NMR (400 MHz, CDCl_3_, *δ*): 1.27 (t, 3H, C*H*_3_–CH_2_–N–); 1.94 (s, 3H, CH_2_=C(C*H*_3_)–COO–); 3.56 (q, 2H, CH_3_–C*H*_2_–N–); 3.75 (t, 2H, –N–C*H*_2_–); 4.39 (t, 2H, –C*H*_2_–OOC–); 5.60 (s, 1H, C*H*_2_=C(CH_3_)–, *trans*); 6.11 (s, 1H, C*H*_2_=C(CH_3_)–, *cis*); 6.84 (d, 2H, H_Ar_(h)); 7.93 (d, 2H, H_Ar_(j)); 7.94 (d, 2H, H_Ar_(k)); 8.33 (d, 2H, H_Ar_(i)); ^13^C NMR (100 MHz, CDCl_3_, *δ*): 12.44 (*C*H_3_–CH_2_–N–); 18.49 (CH_2_=C(*C*H_3_)–COO–); 45.83 (CH_3_–*C*H_2_–N–); 48.95 (–N–*C*H_2_–); 61.84 (–*C*H_2_–OOC–); 111.71 (C_Ar_(g)); 122.77 (C_Ar_(i)); 124.85 (C_Ar_(j)); 126.42 (C_Ar_(h)); 126.53 (*C*H_2_=C(CH_3_)–); 136.06 (C_Ar_(k)); 143.95 (C_Ar_(n)); 147.59 (C_Ar_(p)); 151.49 (C_Ar_(m)); 156.78 (C_Ar_(o)); 167.45 (–*C*OO–); Anal. calcd for C_20_H_22_N_4_O_4_: C 62.82, H 5.80, N 14.65; found C 62.84, H 5.89, N 14.36.

#### 2-(Ethyl(4-((4-nitrophenyl) diazenyl)phenyl)amino)ethyl-2-(2,4,4,6,6,8,8-heptamethyl-1,3,5,7,2,4,6,8-tetraoxatetrasilocan-2-yl)propanoate (9)

A solution of D_4_H (5 g, 17.67 mmol), 8 (20 mol% excess to the D_4_H) and 1 wt% Karstedt's catalyst in dry toluene (50 ml) was heated to 110 °C. The reaction mixture was refluxed overnight under argon. It was then concentrated and the residue was purified by column chromatography (heptane : ethyl acetate = 4 : 1) to obtain 9 as dark red powder in 30% yield.

^1^H NMR (400 MHz, CDCl_3_, *δ*): 0.09 (m, 21H, (C*H*_3_)_2_Si–O–; SiC*H*_3_–O–); 0.72 (2d, 1H, Si–C*H*_2_–, *trans*); 1.02 (2d, 1H, Si–C*H*_2_–, *cis*); 1.20 (d, 3H, –CH_2_–CH(C*H*_3_)–); 1.26 (t, 3H, –N–CH_2_–C*H*_3_); 2.60 (m, 1H, –CH_2_–C*H*(CH_3_)–); 3.55 (q, 2H, –N–C*H*_2_–CH_3_); 3.69 (t, 2H, –N–C*H*_2_–); 4.28 (m, 2H, –C*H*_2_–OOC–); 6.82 (d, 2H, H_Ar_(j)); 7.92 (d, 2H, H_Ar_(l)); 7.93 (d, 2H, H_Ar_(n)); 8.33 (d, 2H, H_Ar_(k)); ^13^C NMR (100 MHz, CDCl_3_, *δ*): 0.25 (Si*C*H_3_–O–); 0.92 ((*C*H_3_)_2_Si–O–); 12.44 (–CH_2_–CH(*C*H_3_)–); 19.51 (–N–CH_2_–C*H*_3_); 21.67 (Si–*C*H_2_–); 34.85 (–CH_2_–*C*H(CH_3_)–); 45.86 (–N–C*H*_2_–CH_3_); 48.99 (–N–*C*H_2_–); 61.31 (–*C*H_2_–OOC–); 111.70 (C_Ar_(j)); 122.75 (C_Ar_(l)); 124.85 (C_Ar_(k)); 126.57 (C_Ar_(m)); 143.92 (C_Ar_(p)); 147.58 (C_Ar_(s)); 151.51 (C_Ar_(o)); 156.75 (C_Ar_(r)); 177.63 (–*C*OO–); MS (ESI) *m*/*z* calc. for C_27_H_44_N_4_O_8_Si_4_: 665.01 [M]^+^, 688.00 [M + Na]^+^; found: 665.117 [M]^+^, 687.098 [M + Na]^+^; Anal. calcd for C_27_H_44_N_4_O_8_Si_4_: C 48.77, H 6.67, N 8.42; found C 49.03, H 6.72, N 8.44.

##### Crystal data for 9

C_27_H_44_N_4_O_8_Si_4_, Mr = 665.02 g mol^−1^, size 0.40 × 0.30 × 0.02 mm^3^, triclinic, space group *P*1̄, *a* = 7.1467(8) Å, *b* = 8.3552(7) Å, *c* = 30.714(2) Å, *α* = 94.797(6)°, *β* = 90.714(2)°, *γ* = 98.677(8)°, *V* = 1806.3(3) Å^3^, *Z* = 2, *ρ*_calcd_ = 1.223 g cm^−3^, *μ*(MoKα) = 0.212 mm^−1^, *F*(000) = 708, 11 991 reflections in *h*(−8/8), *k*(−8/9), *l*(−35/35), measured in the range 4.0 ≤ *Θ* ≤ 48.82, *T* = 293 K, completeness *Θ*_max_ = 100.0%, 5933 independent reflections, *R*_int_ = 0.0452, 350 parameters, 104 restraints, *R*_1obs_ = 0.0947, w*R*_2obs_ = 0.2021, *R*_1all_ = 0.1400, w*R*_2all_ = 0.2282, GoF = 1.095, largest difference peak and hole: 0.68/−0.23 e A^−3^.

#### Synthesis of polydimethylsiloxanes P-MNA and P-MDR1

A solution of tetramethylammonium hydroxide solution 25% in methanol (TMAH) (4.6 μl) was dried by azeotropic distillation in benzene. Subsequently, the monomer 6 or 9 (5 g) was added and the reaction mixture was stirred to 100 °C for 24 h. Then, the temperature was raised to 140 °C for 1 h to decompose the initiator. The reaction mixture was washed several times and precipitated twice in THF/methanol in order to remove unreacted cycles and oligomers.

##### P-MNA

^1^H NMR (400 MHz, CDCl_3_, *δ*): 0.06 (21.53H, (C*H*_3_)_2_Si–O–; SiC*H*_3_–O–); 0.65 (1H, Si–C*H*_2_–, *trans*); 0.95 (1H, Si–C*H*_2_–, *cis*); 1.13 (3H, –CH_2_–CH(C*H*_3_)–); 2.53 (3H, –CH_2_–C*H*(CH_3_)–); 3.10 (3H, C*H*_3_–N–); 3.70 (2H, –N–C*H*_2_–); 4.25 (2H, –C*H*_2_–OOC–); 6.66 (2H, H_Ar_(i)); 8.08 (2H, H_Ar_(j)); ^13^C NMR (100 MHz, CDCl_3_, *δ*): 0.50 (Si*C*H_3_–O–); 1.20 ((*C*H_3_)_2_Si–O–); 19.40 (–CH_2_–CH(*C*H_3_)–); 21.91 (Si–*C*H_2_–); 34.75 (–CH_2_–*C*H(CH_3_)–); 39.09 (*C*H_3_–N–); 50.92 (–N–*C*H_2_–); 60.96 (–*C*H_2_–OOC–); 110.57 (C_Ar_(k)); 126.25 (C_Ar_(l)); 137.51 (C_Ar_–NO_2_); 153.51 (C_Ar_–N); 177.38 (–*C*OO–); Anal. calcd for C_20_H_38_N_2_O_8_Si_4_: C 43.93, H 7.00, N 5.12; found C 43.81, H 7.07, N 5.20; GPC: *M*_n_ = 36 578 g mol^−1^; *M*_w_ = 95 962 g mol^−1^; PDI = 2.62.

##### P-MDR1

^1^H NMR (400 MHz, CDCl_3_, *δ*): 0.07 (21.76H, (C*H*_3_)_2_Si–O– and –SiC*H*_3_–O–); 0.71 (1H, Si–C*H*_2_–, *trans*); 1.01 (1H, Si–C*H*_2_–, *cis*); 1.20 (6H, –CH_2_–CH(C*H*_3_)–; –N–CH_2_–C*H*_3_); 2.59 (1H, –CH_2_–C*H*(CH_3_)–); 3.51 (2H, –N–C*H*_2_–CH_3_); 3.64 (2H, –N–C*H*_2_–); 4.27 (2H, –C*H*_2_–OOC–); 6.78 (2H, H_Ar_(j)); 7.90 (4H, H_Ar_(l) and H_Ar_(n)); 8.28 (2H, H_Ar_(k)); ^13^C NMR (100 MHz, CDCl_3_, *δ*): 0.55 (Si*C*H_3_–O–); 1.21 ((*C*H_3_)_2_Si–O–); 12.44 (–CH_2_–CH(*C*H_3_)–); 19.51 (–N–CH_2_–C*H*_3_); 21.97 (Si–*C*H_2_–); 34.79 (–CH_2_–*C*H(CH_3_)–); 45.83 (–N–C*H*_2_–CH_3_); 48.93 (–N–*C*H_2_–); 61.24 (–*C*H_2_–OOC–); 111.59 (C_Ar_(j)); 122.74 (C_Ar_(l)); 124.80 (C_Ar_(k)); 126.45 (C_Ar_(m)); 143.93 (C_Ar_(p)); 147.56 (C_Ar_(s)); 151.37 (C_Ar_(o)); 156.76 (C_Ar_(r)); 177.29 (–*C*OO–); Anal. calcd for C_27_H_44_N_2_O_8_Si_4_: C 48.77, H 6.67, N 8.42; found C 48.64, H 6.75, N 8.67; GPC: *M*_n_ = 22 624 g mol^−1^; *M*_w_ = 78 607 g mol^−1^; PDI = 3.47.

## Conclusions

We have developed a synthetic strategy to tetracyclosiloxane monomers that carry push–pull moieties and showed that it is possible to polymerize them by ring opening polymerization in presence of tetramethylammoinum hydroxide to polymers with appreciable molar masses. The content of push–pull groups can be tuned by copolymerizing a mixture of octamethyltetracyclosiloxane and push–pull functionalized cyclosiloxane monomers. Due to the high flexibility of the polysiloxy backbone, the *T*_g_ of all polymers that carry a nitroaniline group is well below RT. However, the polysiloxane modified with Disperse Red 1 group, which has a larger dipole as compared to nitroaniline, showed no *T*_g_. This polymer is solid at RT and melted at 55.4 °C. The highest value of the dielectric permittivity for the polymers modified with nitroaniline was *ε*′ = 17.3 at RT. Because P-MDR1 is solid at RT, its dielectric permittivity is low, since the dipoles are frozen and cannot contribute to permittivity *via* orientation polarization. However, its permittivity value increases to 21, when increasing the temperature above *T*_m_, where the dipoles are mobile. Due to the low *T*_g_ of P-MNA and its high permittivity and large values for *α* and *β* characteristic for low dipolar interactions, this polymer is particularly attractive as dielectric in electromechanical actuators. Further work in this direction is underway.

## Conflicts of interest

There are no conflicts to declare.

## Supplementary Material

RA-008-C8RA00707A-s001

RA-008-C8RA00707A-s002
