# Supplementary material for: Synthesis of novel cyclosiloxane monomers containing push–pull moieties and their anionic ring opening polymerization
Source: RSC Adv. 2018 Feb 16;8(14):7569–78. doi: 10.1039/c8ra00707a (PMC9078476; doi:10.1039/c8ra00707a)
Supplement: RA-008-C8RA00707A-s001 [file RA-008-C8RA00707A-s001.pdf]

Electronic Supplementary Material (ESI) for RSC Advances.
This journal is © The Royal Society of Chemistry 2018

Supporting information

Synthesis of novel cyclosiloxane monomers containing push-pull moieties and their anionic ring opening polymerization

Elena Perju,[a,b] Eduardo Cuervo-Reyes,[c] Sergiu Shova,[b] and Dorina M. Opris[a]*

[a] *Swiss Federal Laboratories for Materials Science and Technology Empa, Laboratory for Functional Polymers, Ueberlandstr. 129, Ch-8600, Dübendorf, Switzerland, Dorina.opris@empa.ch.*

[b] *"Petru Poni" Institute of Macromolecular Chemistry of Romanian Academy, Aleea Gr. Chica Voda, 41A, 700487, Iasi, Romania.*

[c] *Swiss Federal Laboratories for Materials Science and Technology Empa, Laboratory for Energy Conversion, Ueberlandstr. 129, CH-8600, Dübendorf, Switzerland.*

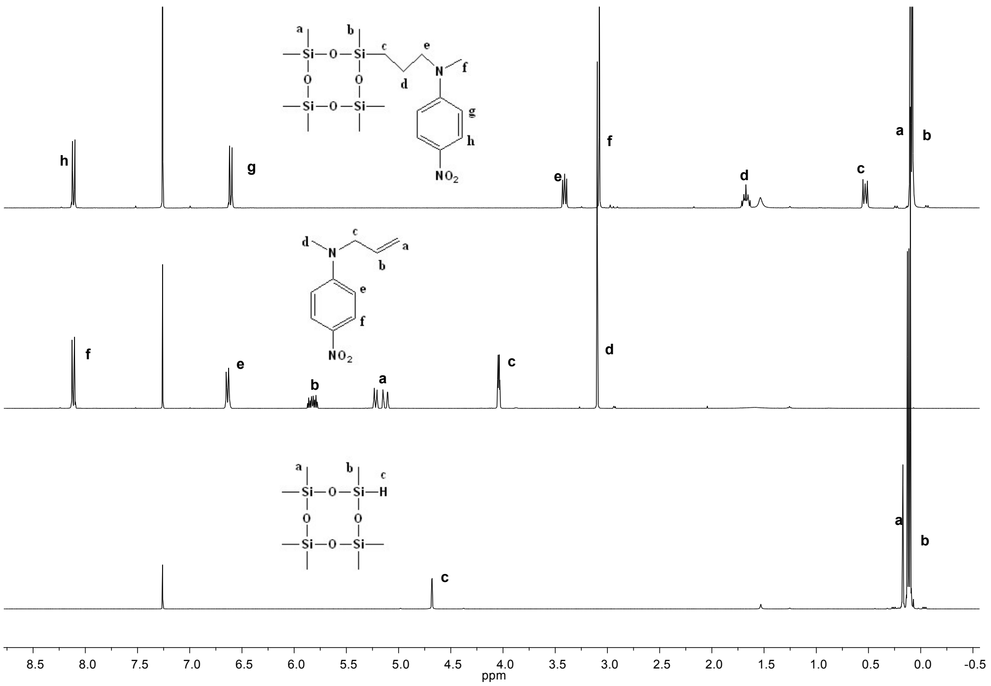

Figure S1: ^1H NMR spectra of D_4H, N-allyl-N-methyl-4-nitroaniline and the cyclosiloxane monomer **3**.

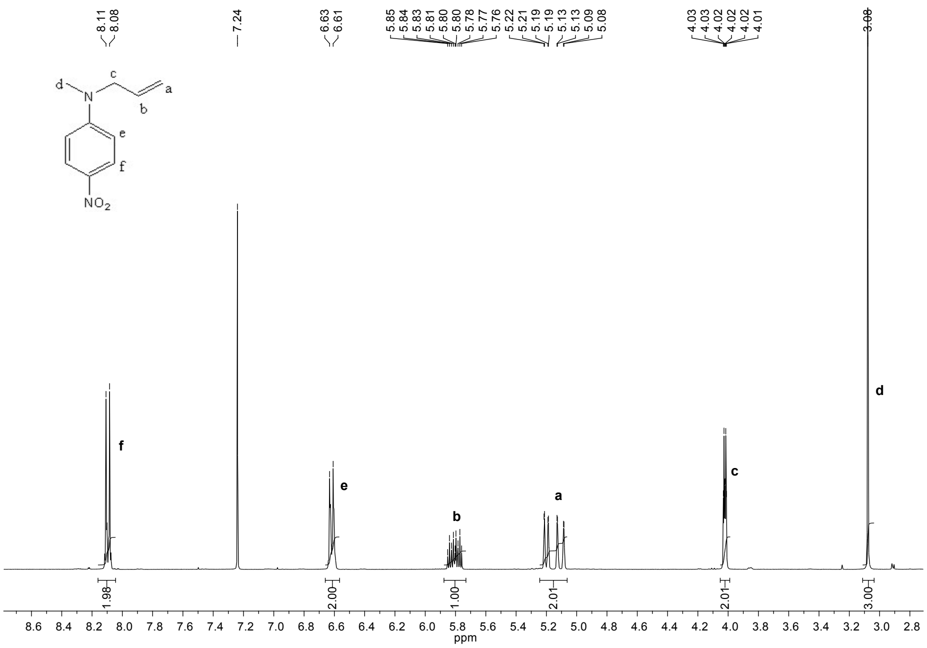

Figure S2: ^{1}H NMR spectrum of N-allyl-N-methyl-4-nitroaniline **2**.

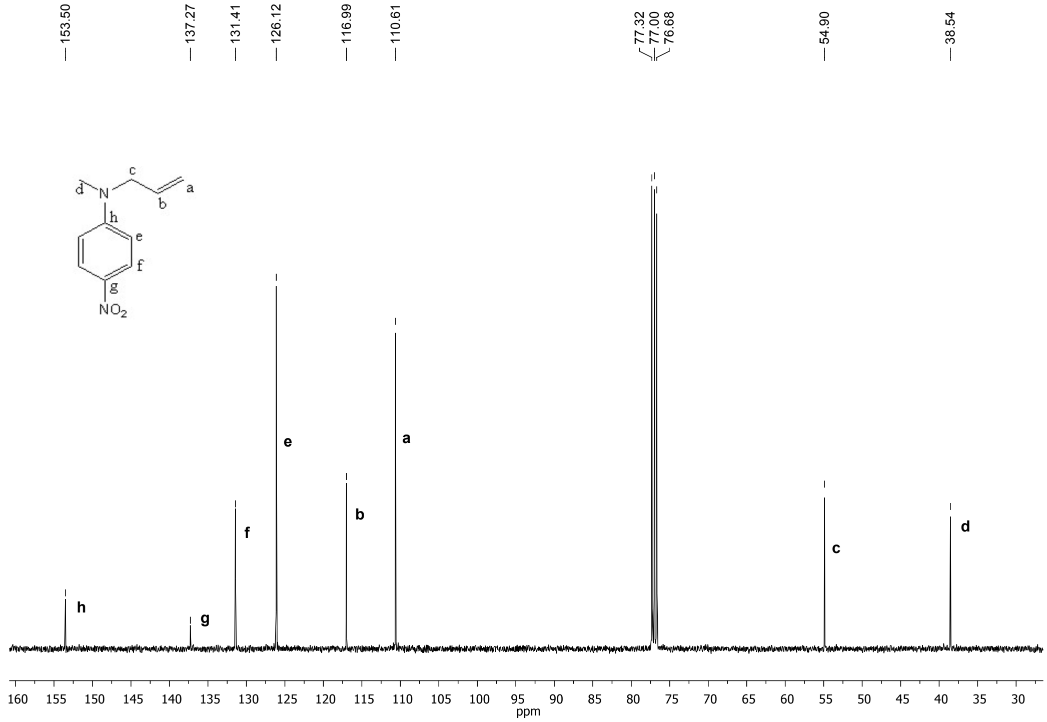

Figure S3: ^{13}C NMR spectrum of N-allyl-N-methyl-4-nitroaniline **2**.

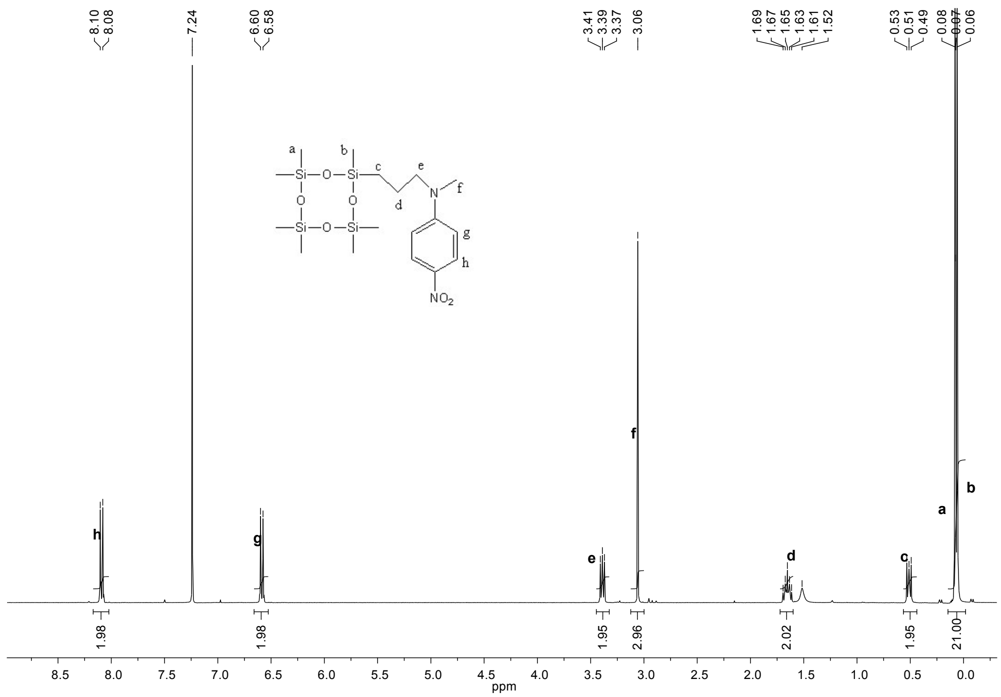

Figure S4: ^{1}H NMR spectrum of **3**.

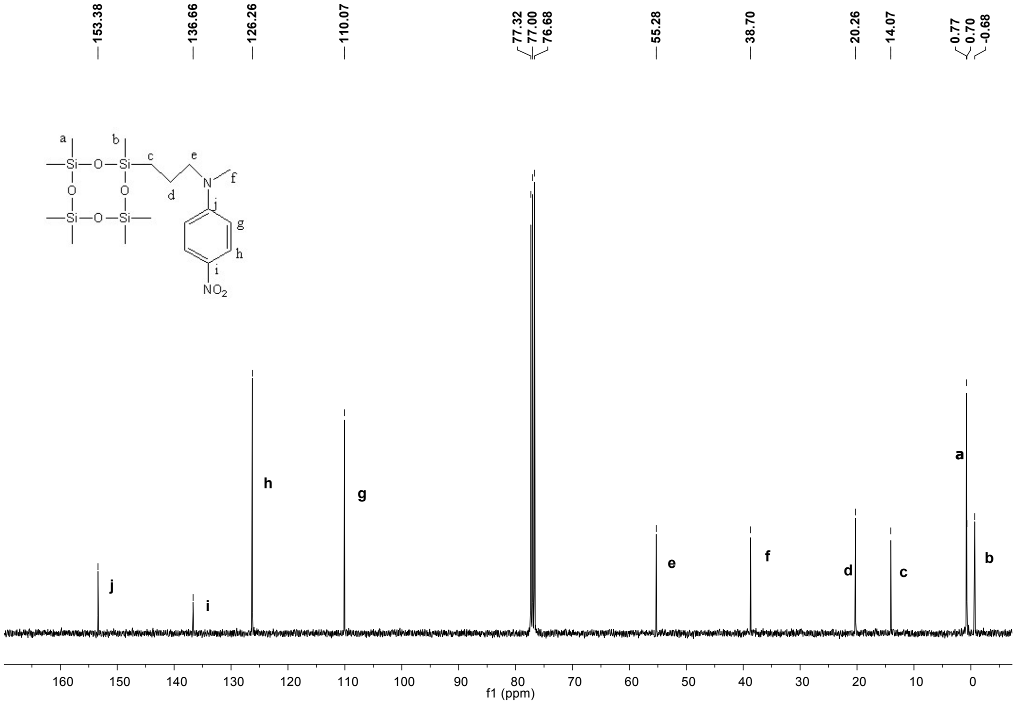

Figure S5: ^{13}C NMR spectrum of **3**.

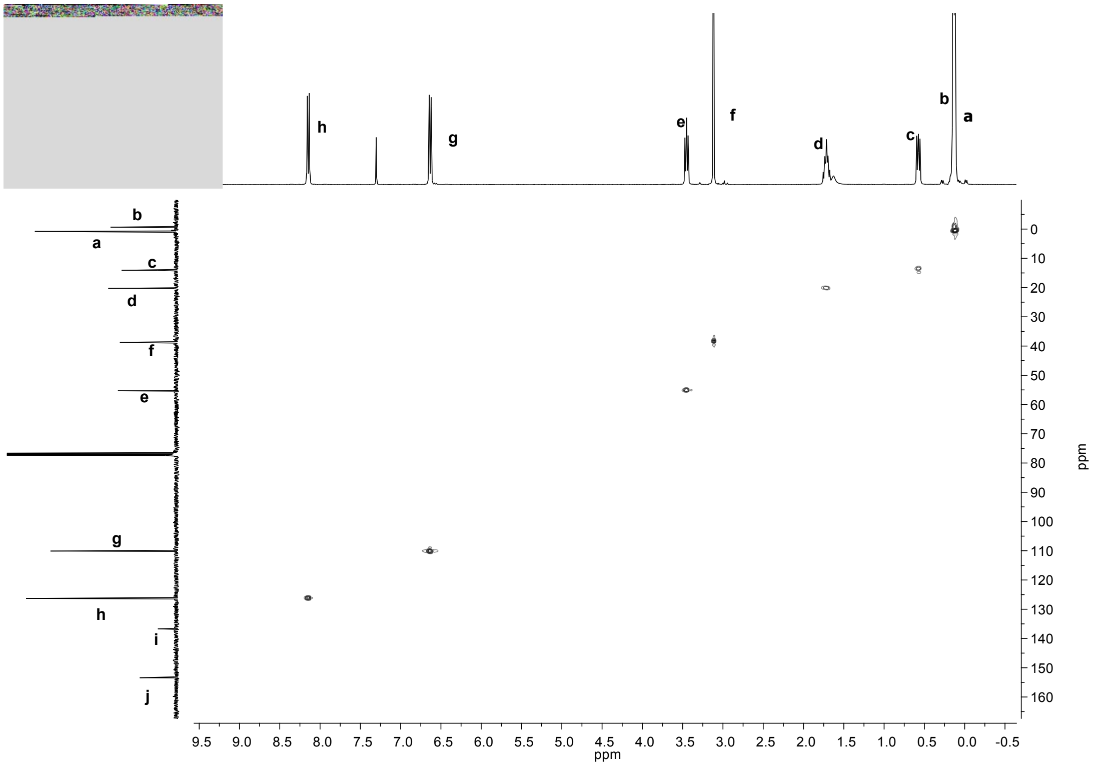

Figure S6: HSQC spectrum of **3**.

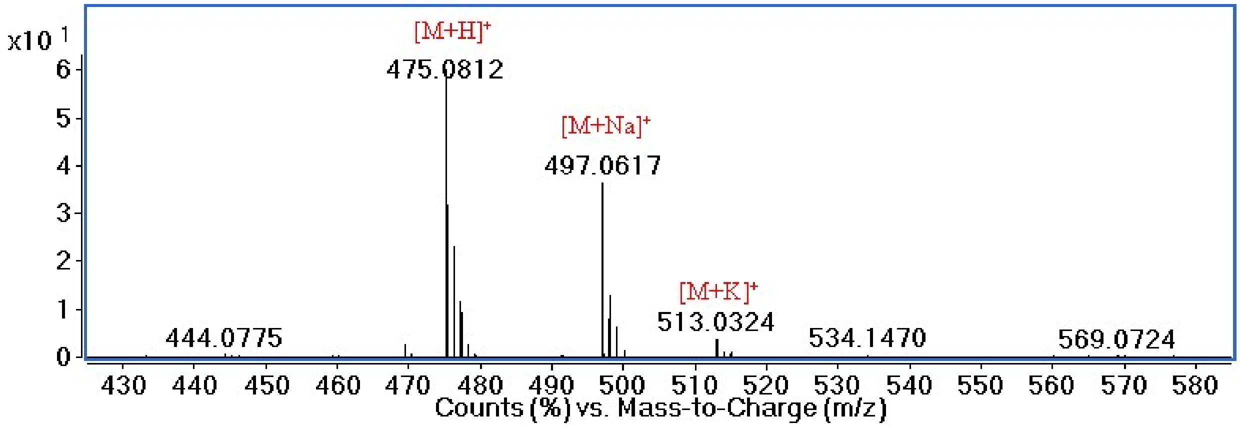

Figure S7: ESI – mass spectrum of **3**.

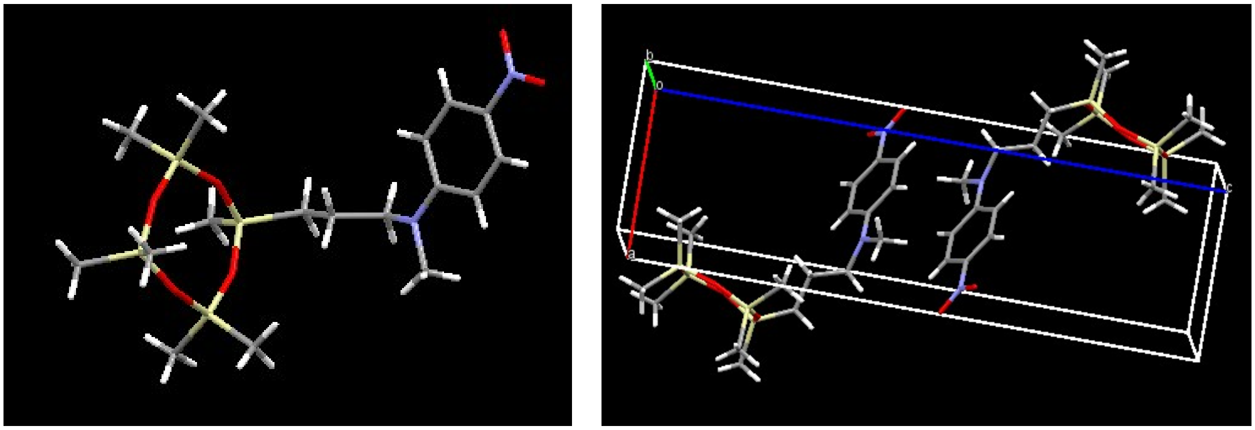

(a) **(b)**

Figure S8: X-ray structure of **3**.

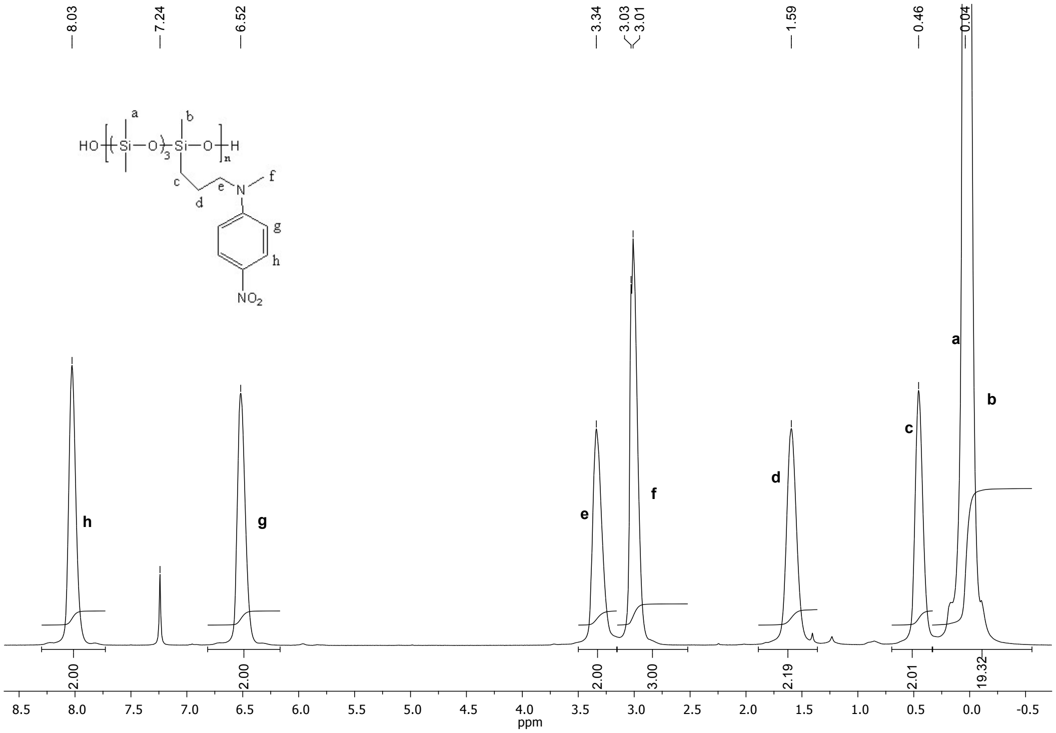

Figure S9: ^{1}H NMR spectrum of **P-NA**.

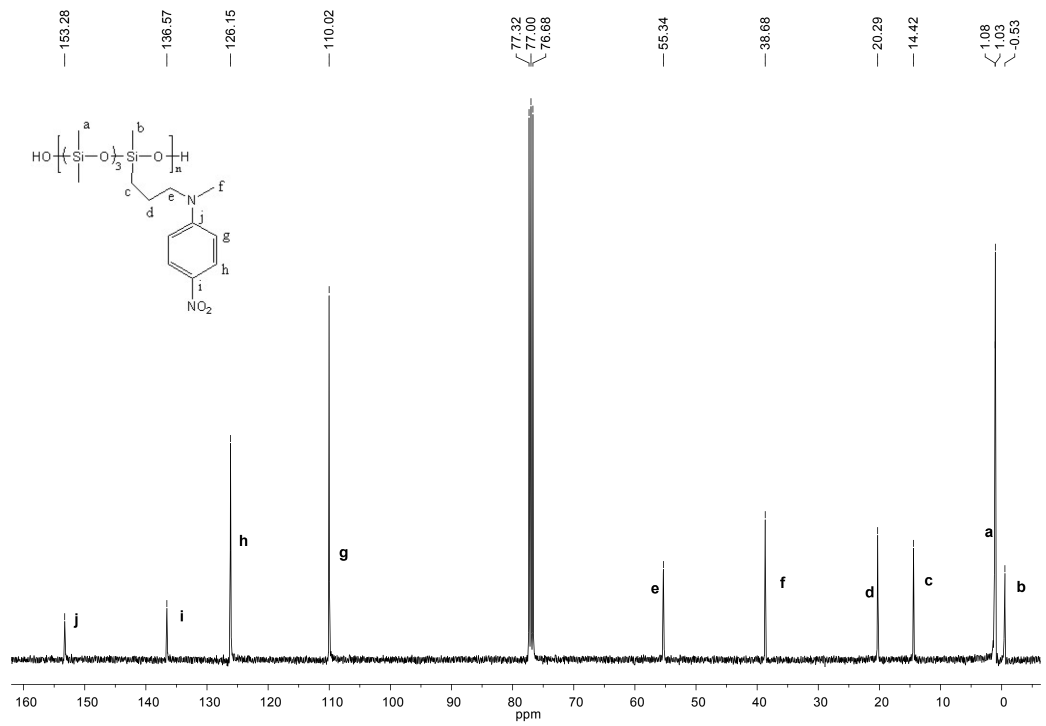

Figure S10: ^{13}C NMR spectrum of **P-NA**.

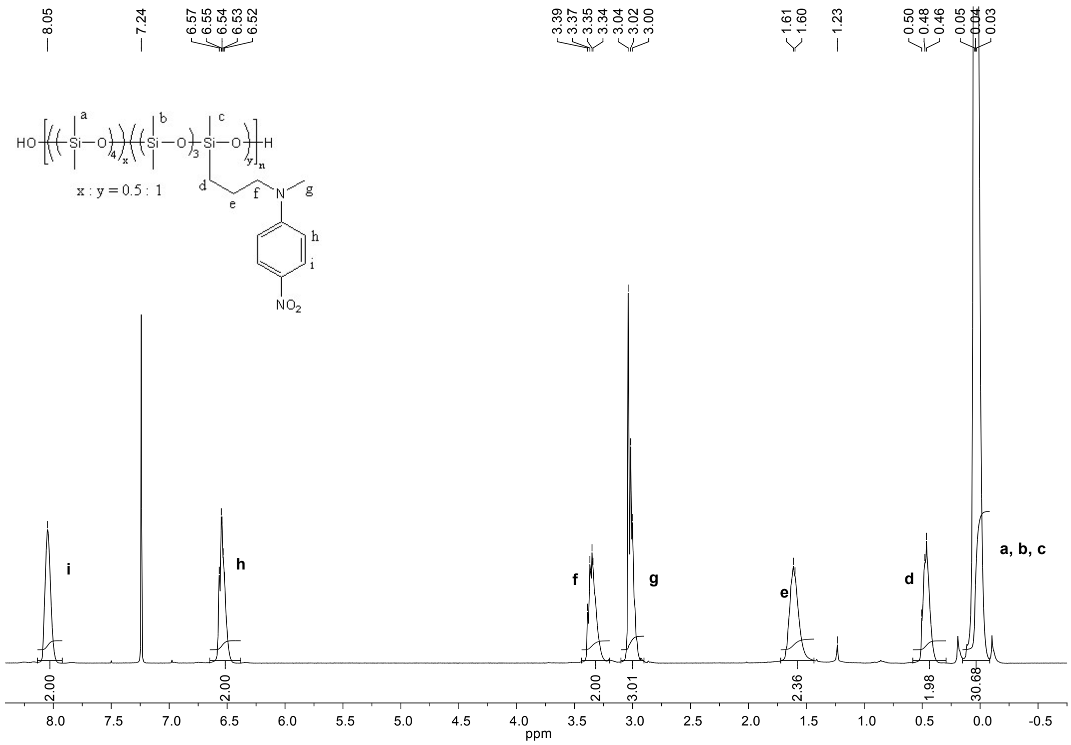

Figure S11: ^{1}H NMR spectrum of ***co*-P-NA (1:2)**.

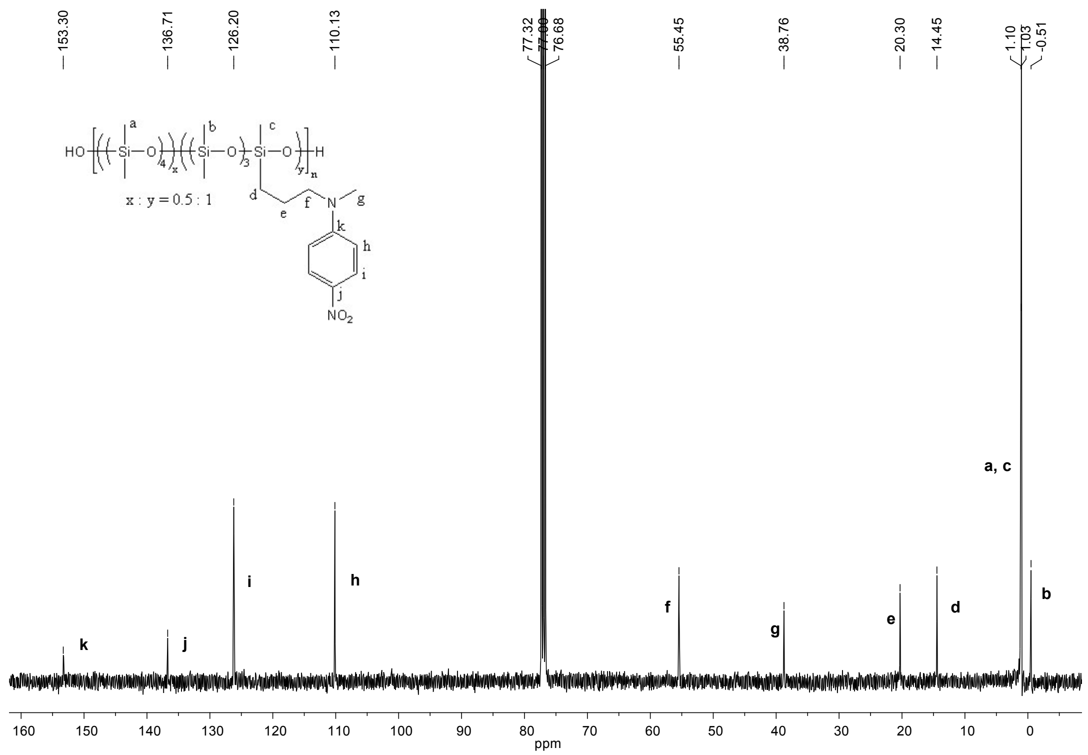

Figure S12: ^{13}C NMR spectrum of ***co*-P-NA (1:2)**.

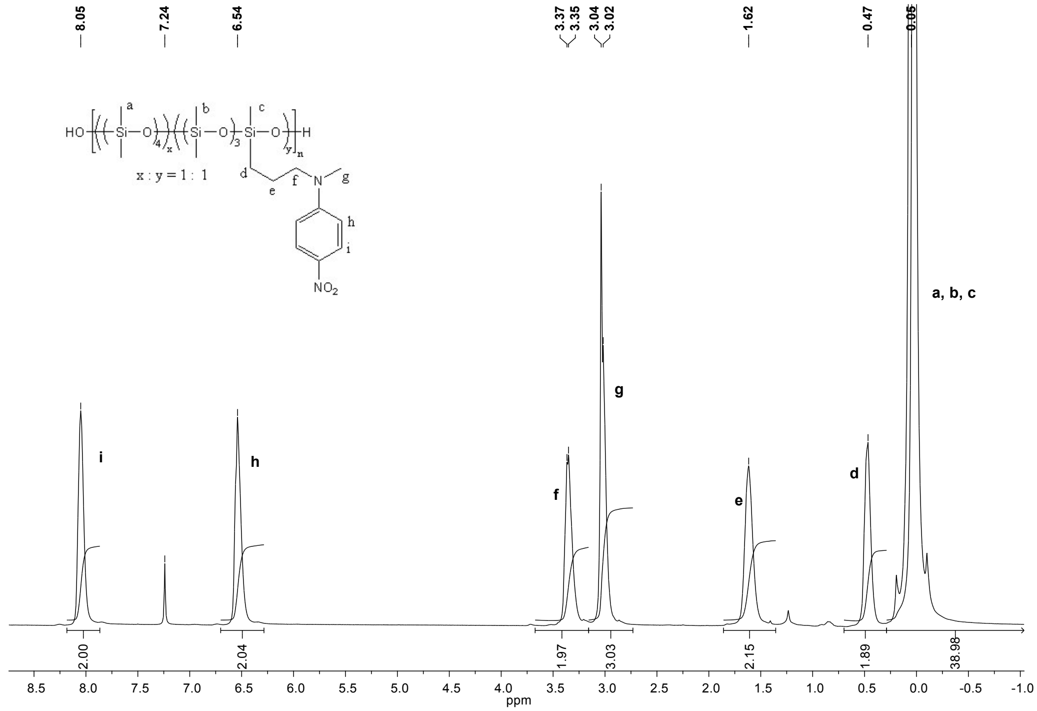

Figure S13: ^{1}H NMR spectrum of ***co*-P-NA (1:1)**.

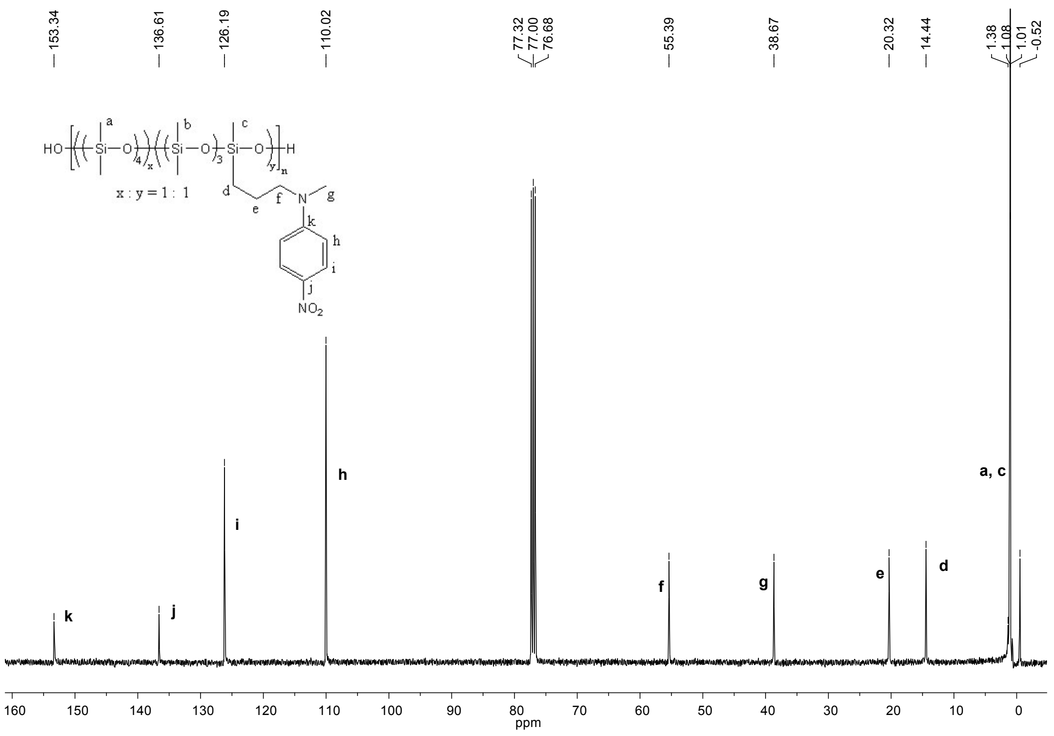

Figure S14: ^{13}C NMR spectrum of ***co*-P-NA (1:1)**.

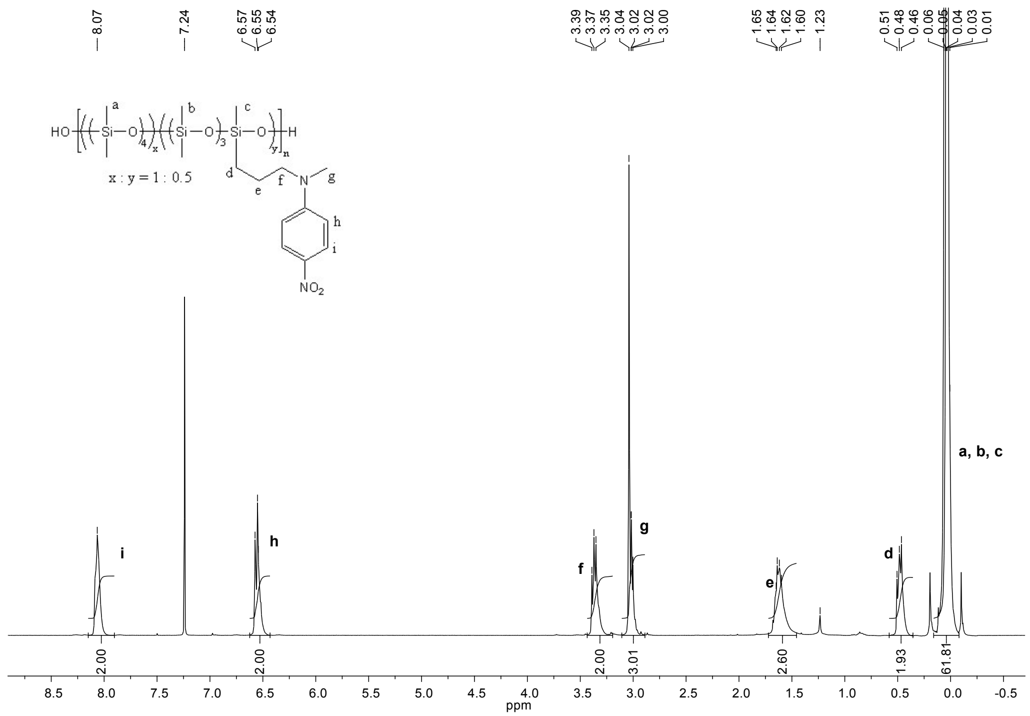

Figure S15: ^{1}H NMR spectrum of ***co*-P-NA (2:1)**.

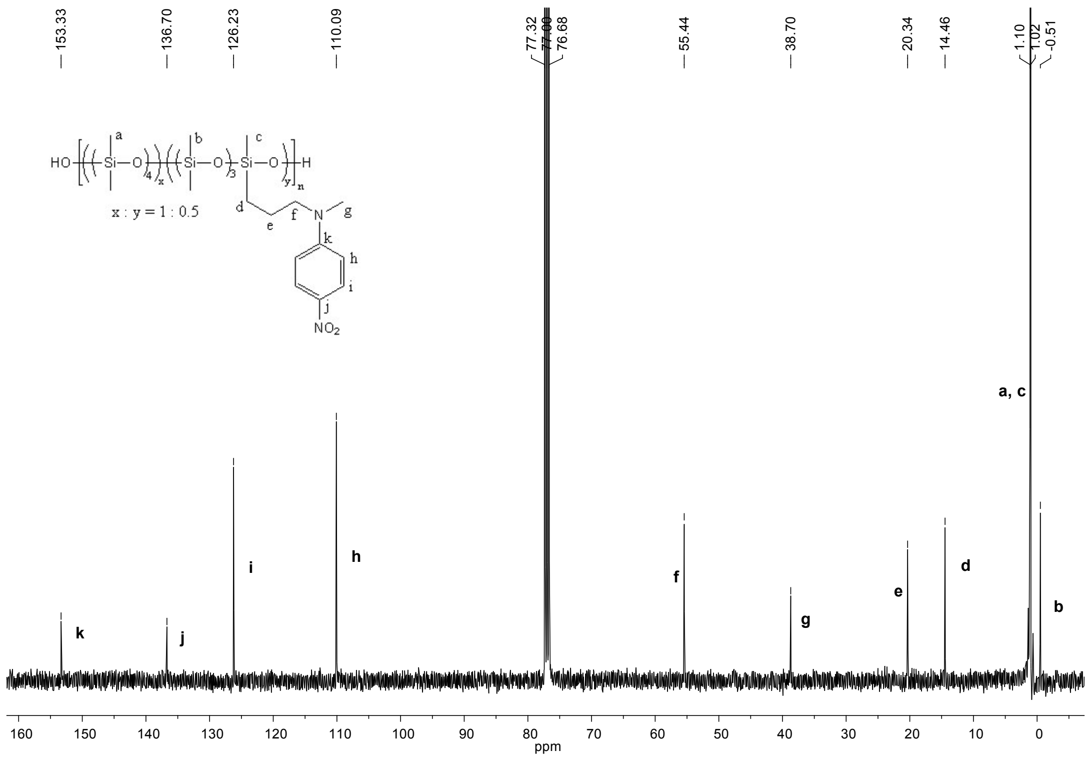

Figure S16: ^{13}C NMR spectrum of ***co*-P-NA (2:1)**.

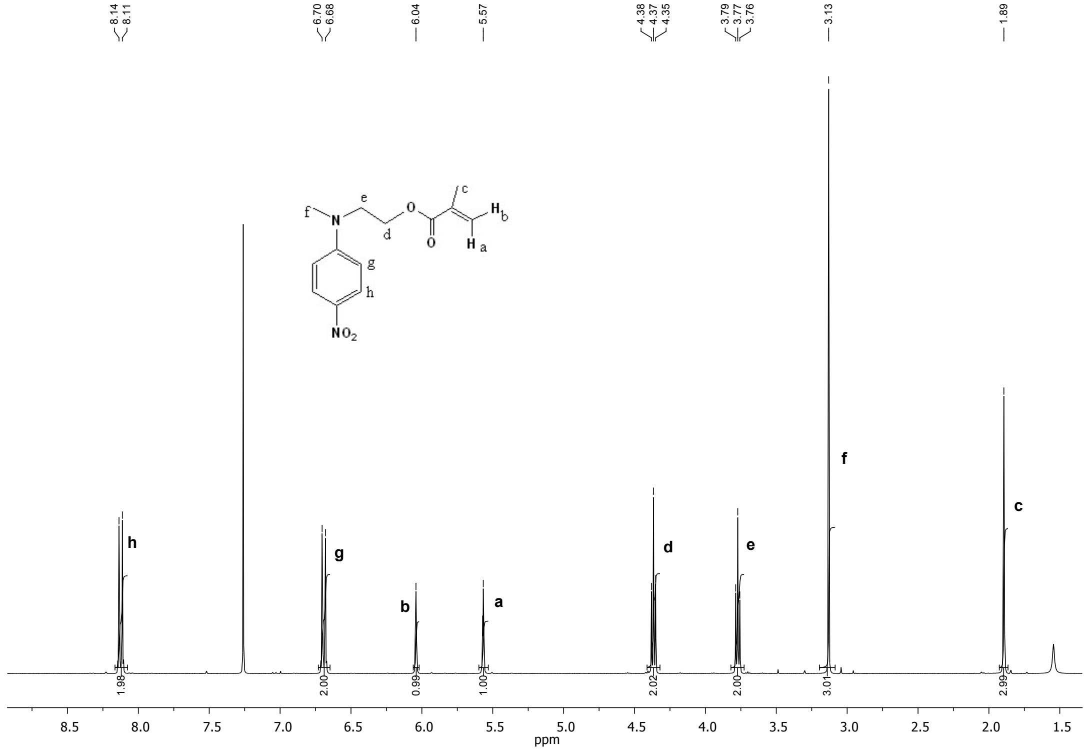

Figure S17: ^{1}H NMR spectrum of **5**.

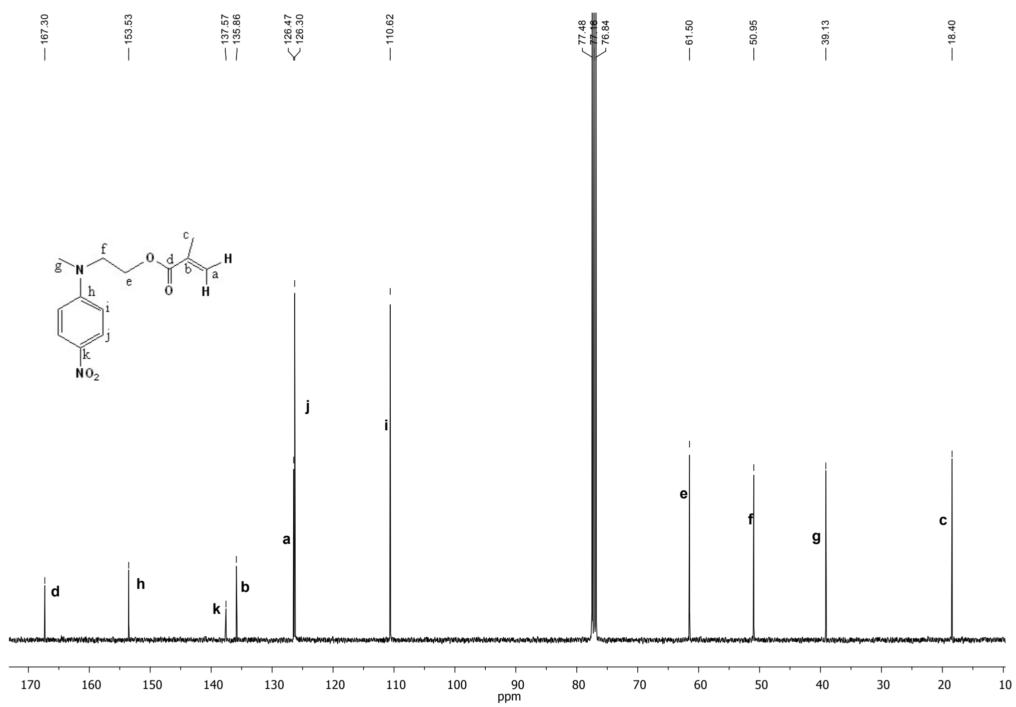

Figure S18: ^{13}C NMR spectrum of **5**.

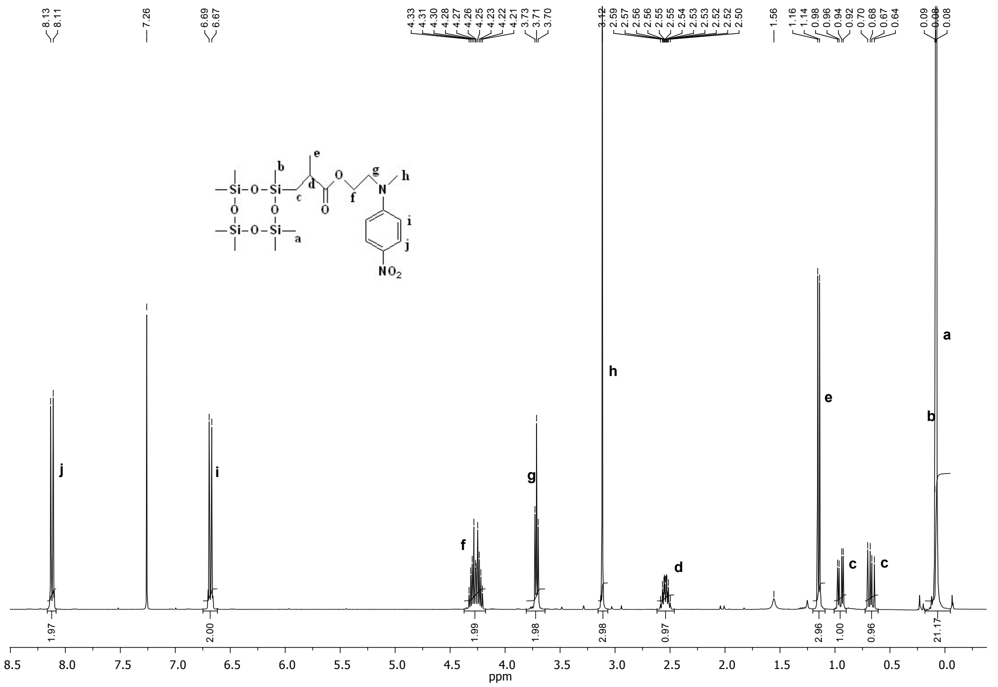

Figure S19: ^{1}H NMR spectrum of **6**.

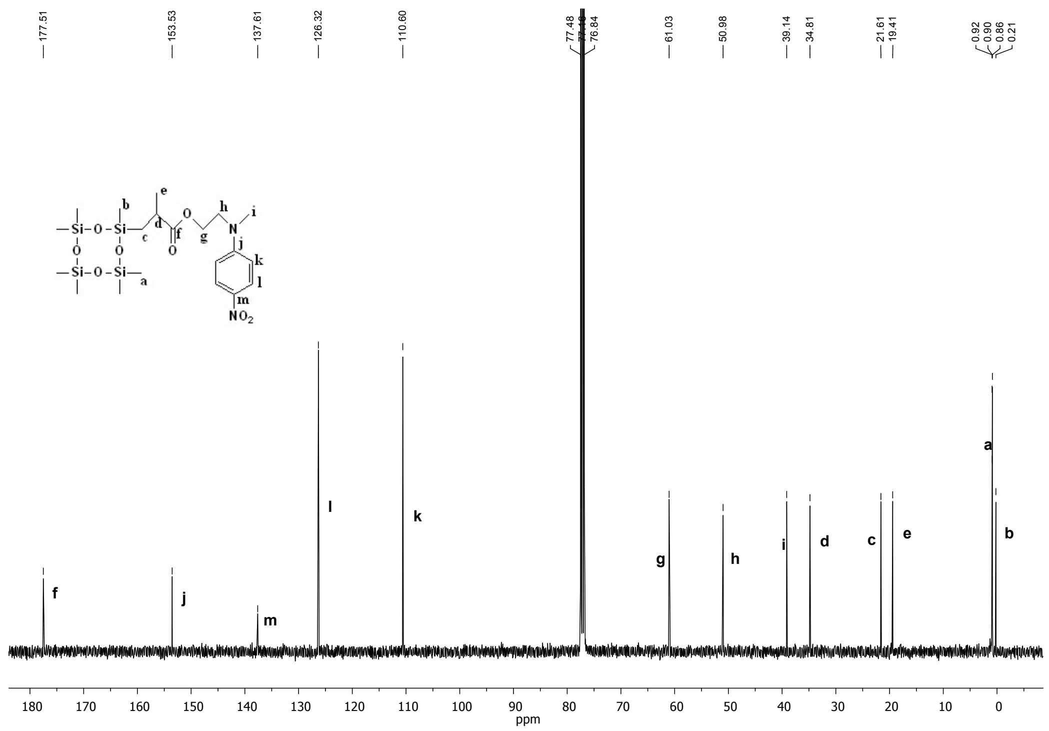

Figure S20: ^{13}C NMR spectrum of **6**.

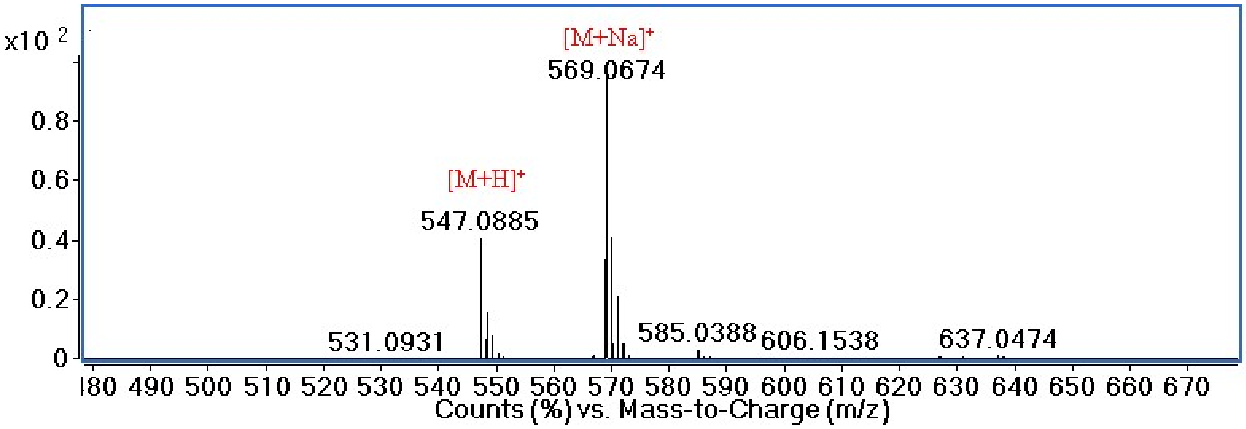

Figure S21: ESI – mass spectrum of **6**.

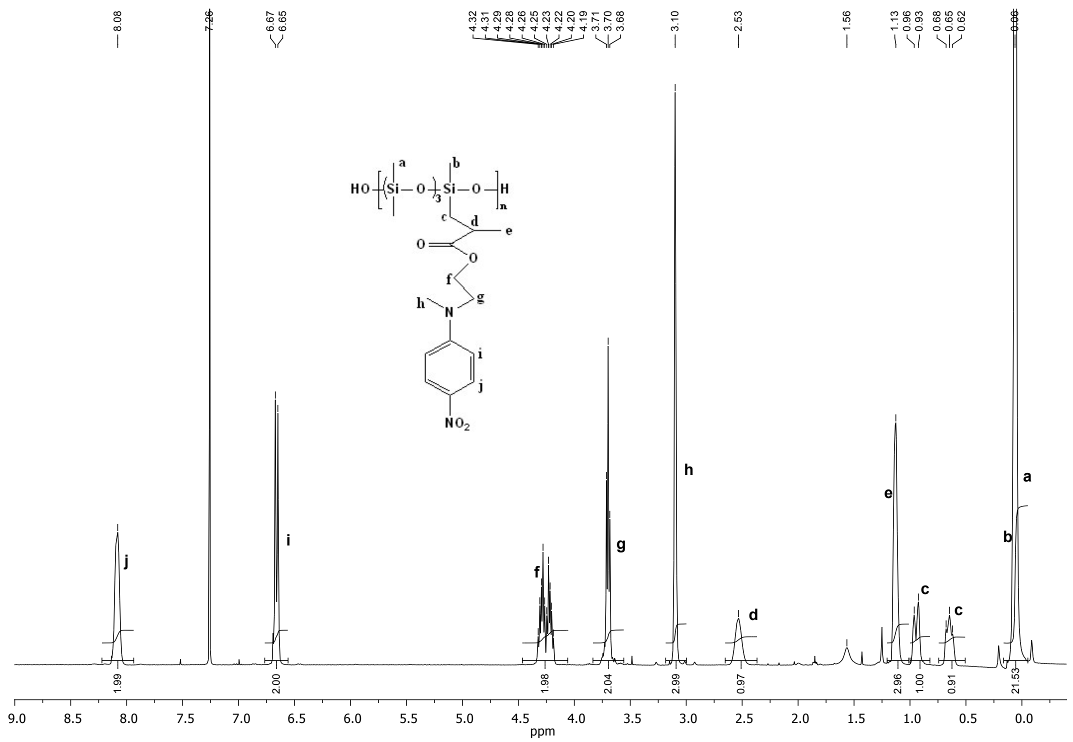

Figure S22: ^{1}H NMR spectrum of **P-MNA**.

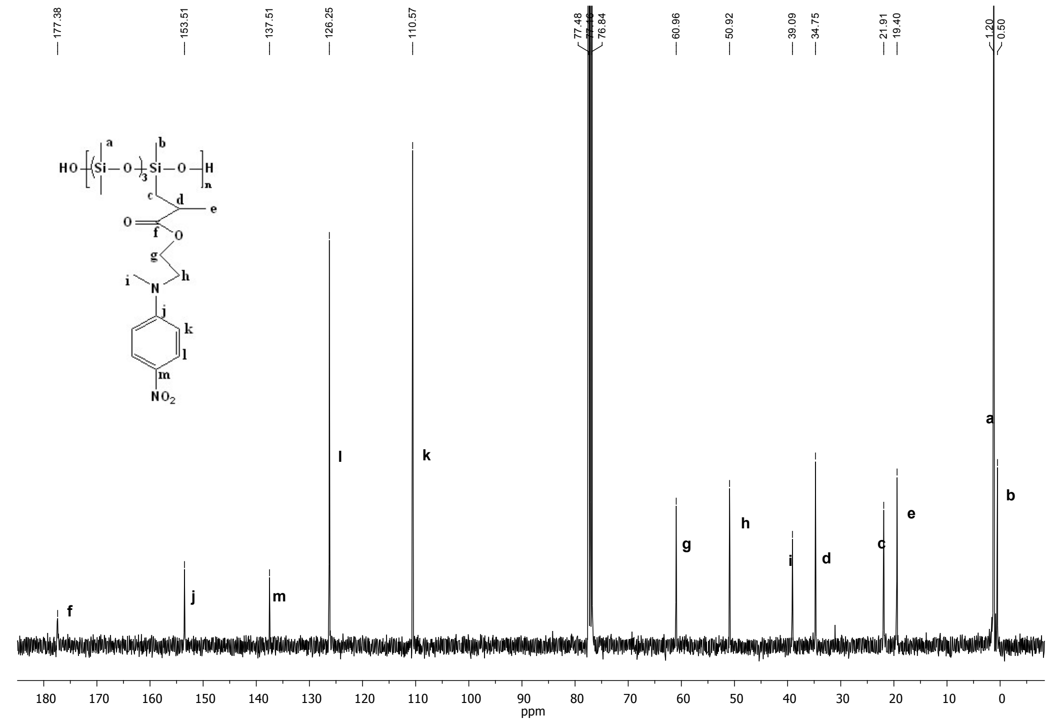

Figure S23: ^{13}C NMR spectrum of **P-MNA**.

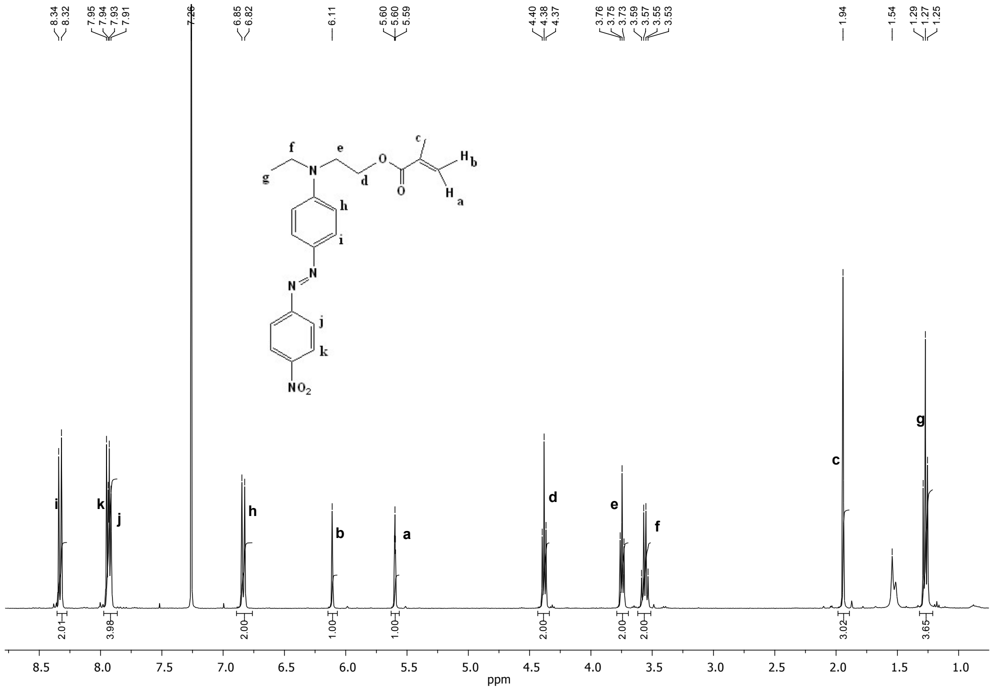

Figure S24: ^{1}H NMR spectrum of **8**.

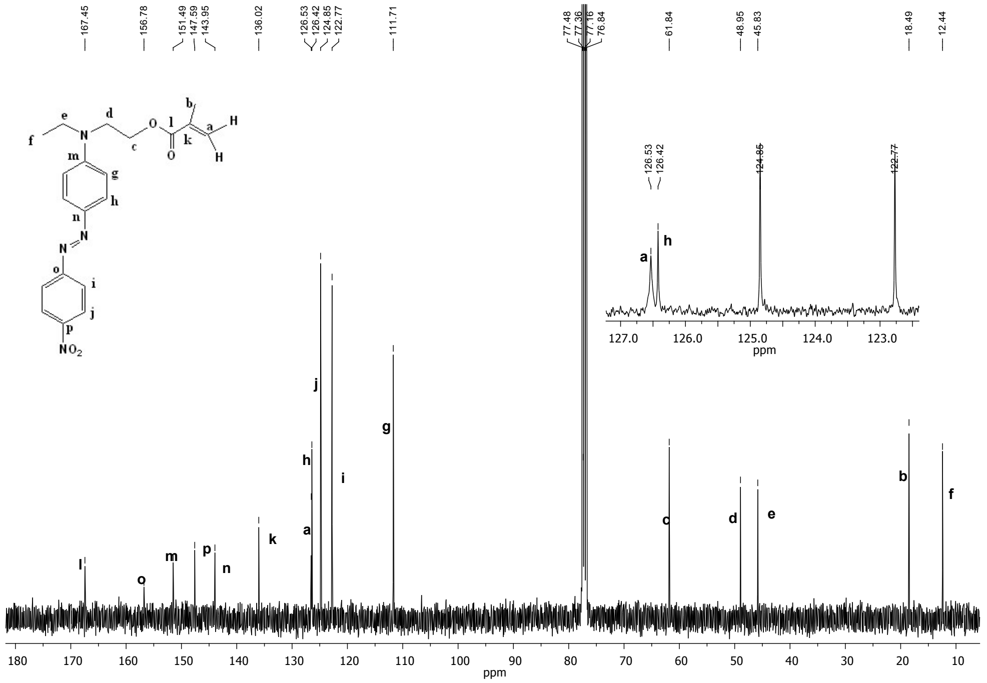

Figure S25: ^{13}C NMR spectrum of **8**.

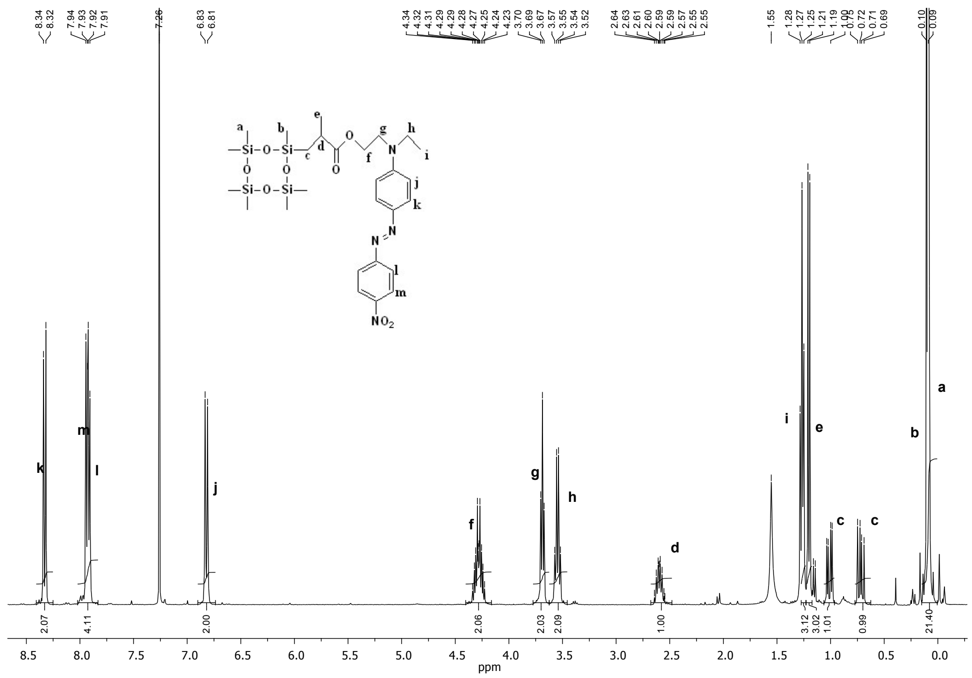

Figure S26: ^{1}H NMR spectrum of **9**.

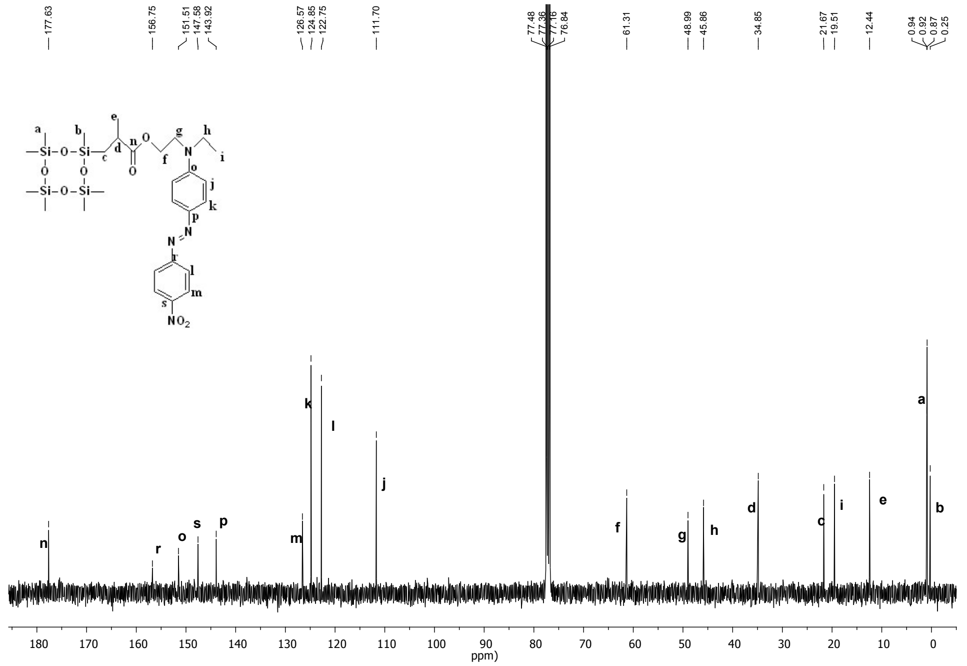

Figure S27: ^{13}C NMR spectrum of **9**.

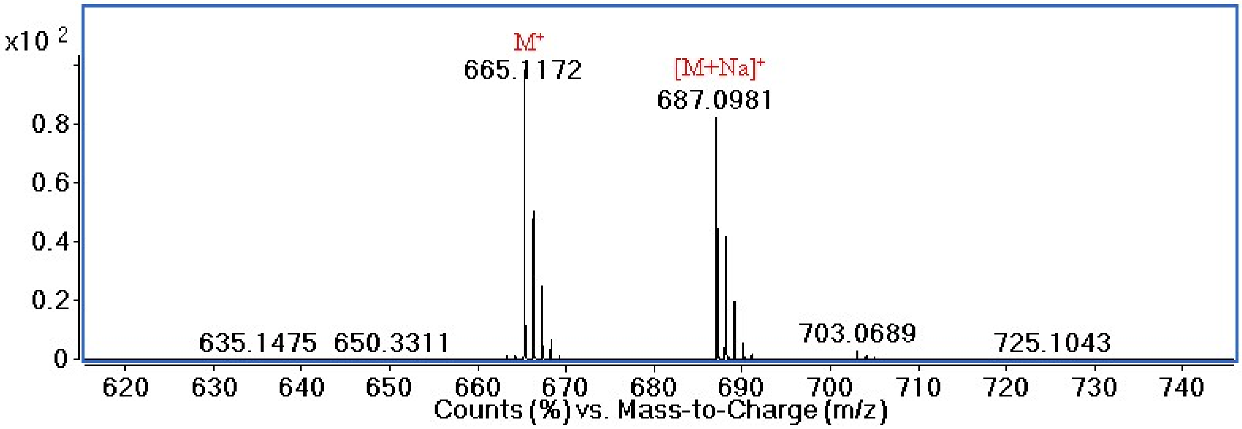

Figure S28: ESI – mass spectrum of **9**.

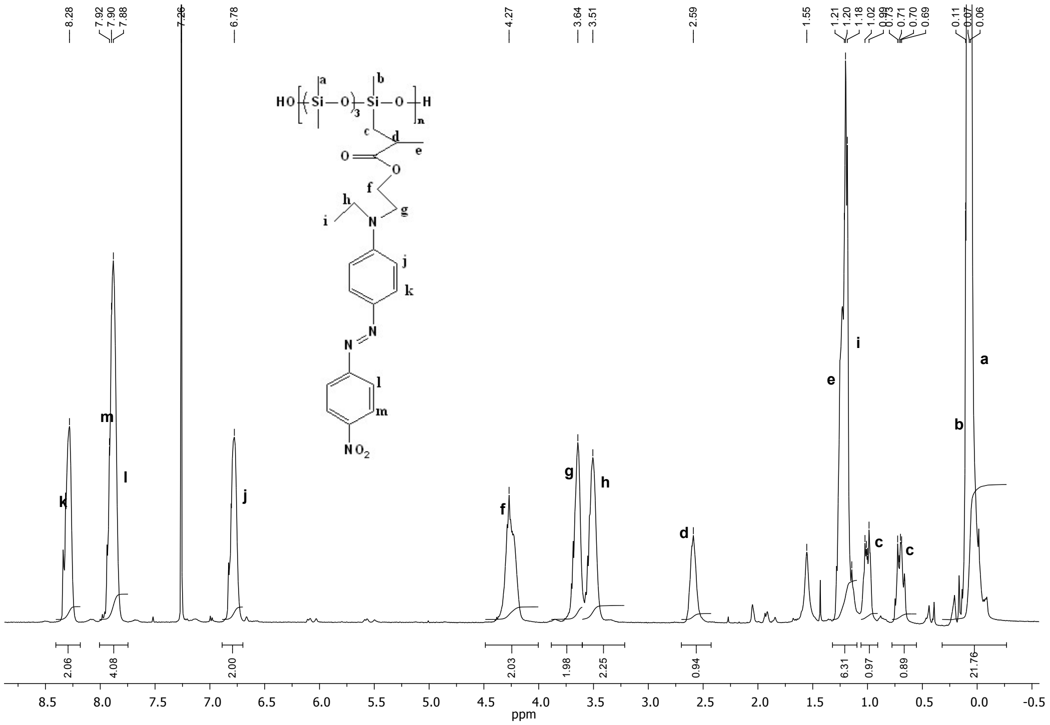

Figure S29: ^{1}H NMR spectrum of **P-MDR1**.

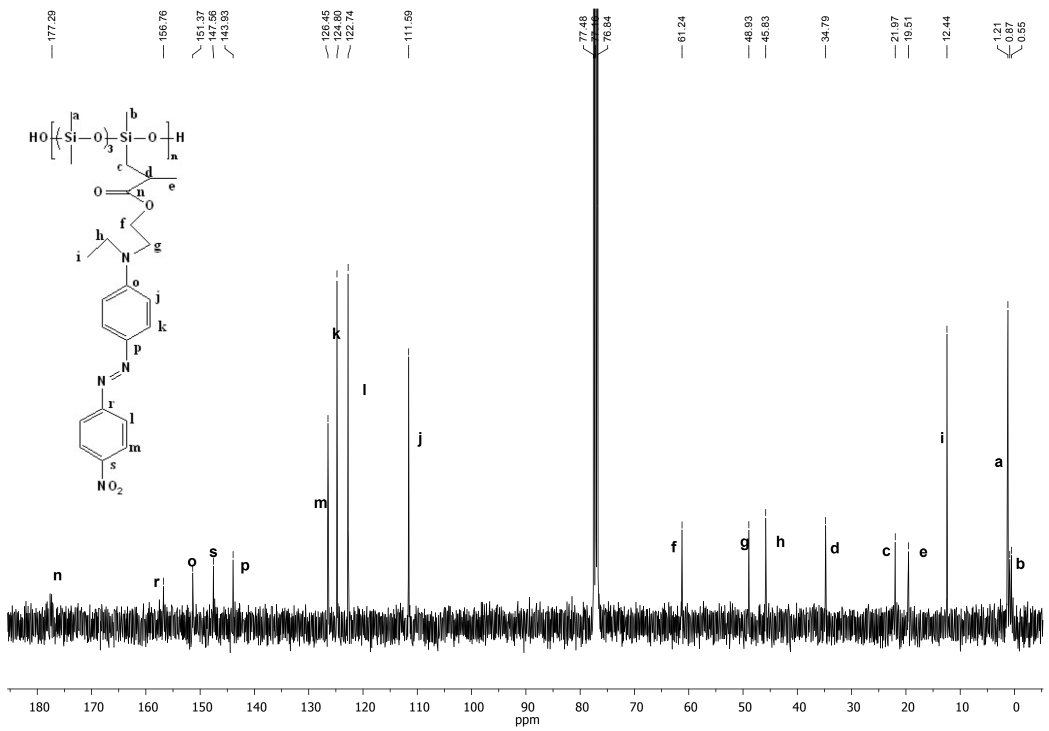

Figure S30: ^{13}C NMR spectrum of **P-MDR1**.

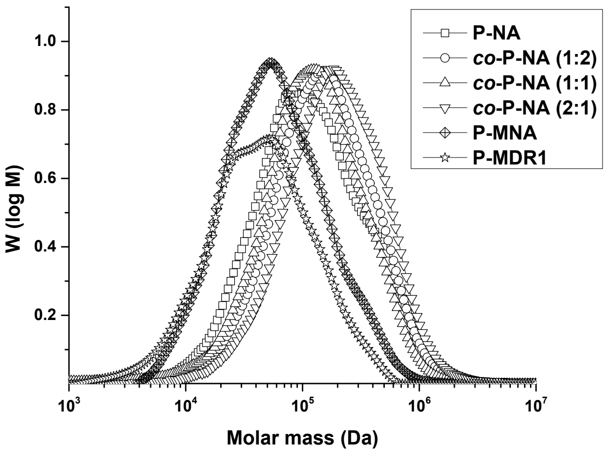

Figure S31. GPC elugrams of **P-NA**, ***co*-P-NA(x:y)**, **P-MNA**, and **P-MDR1** in THF.

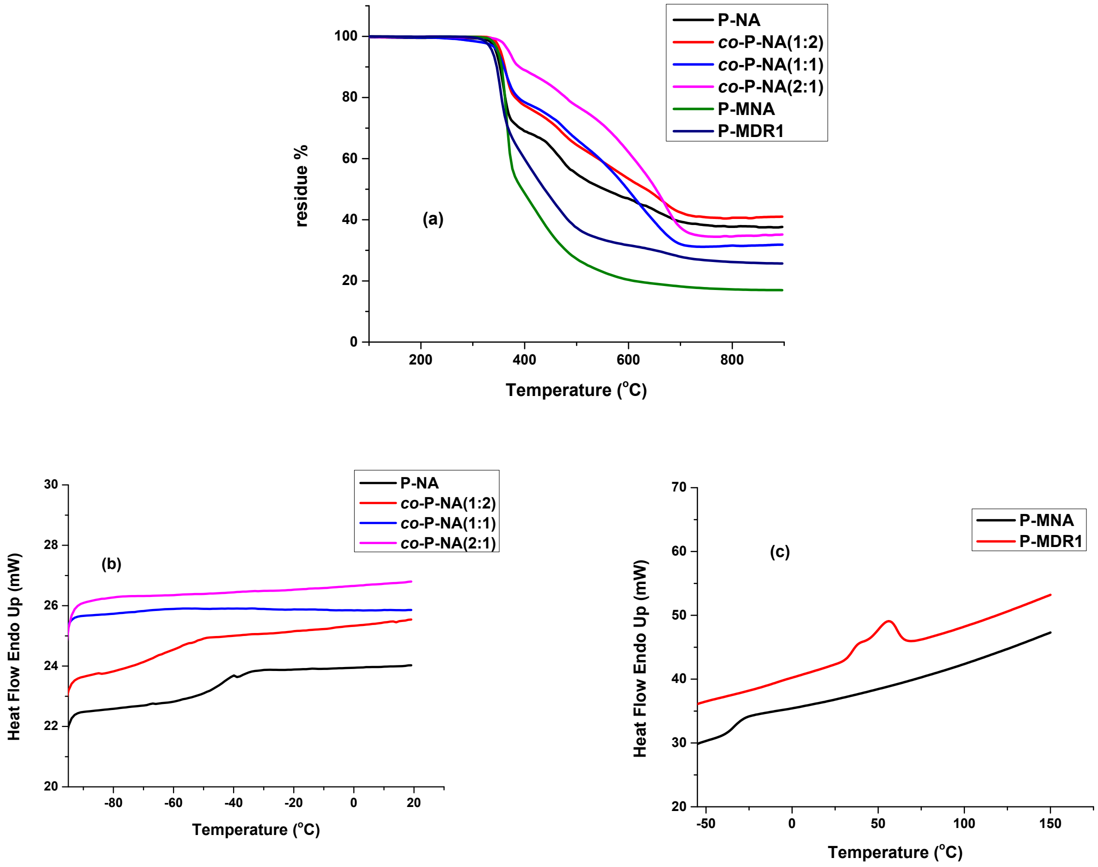

Figure S32. TGA (a) and DSC (b,c) traces of **P-NA**, ***co*-P-NA(x:y)**, **P-MNA**, and **P-MDR1**.

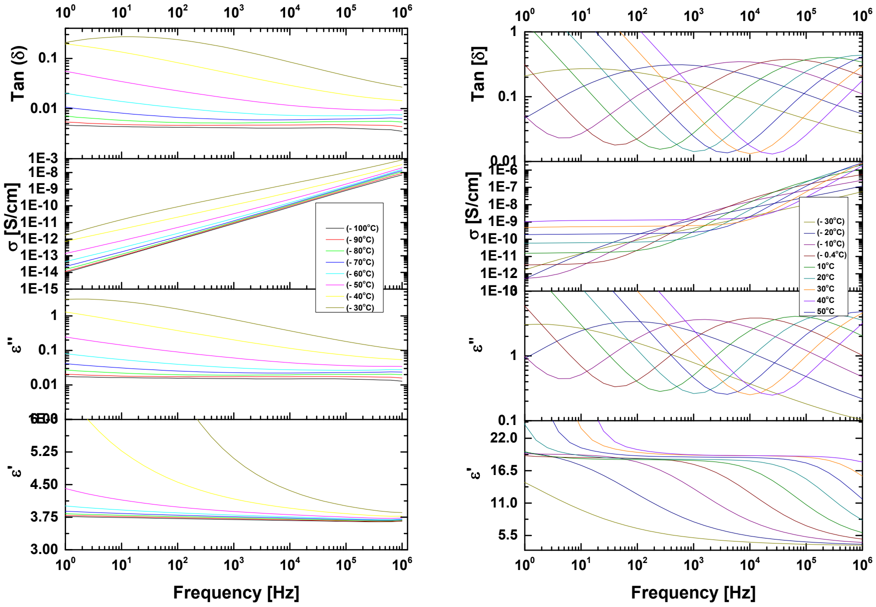

Figure S33: Dielectric properties of the **P-NA** as function of frequency at different temperature.

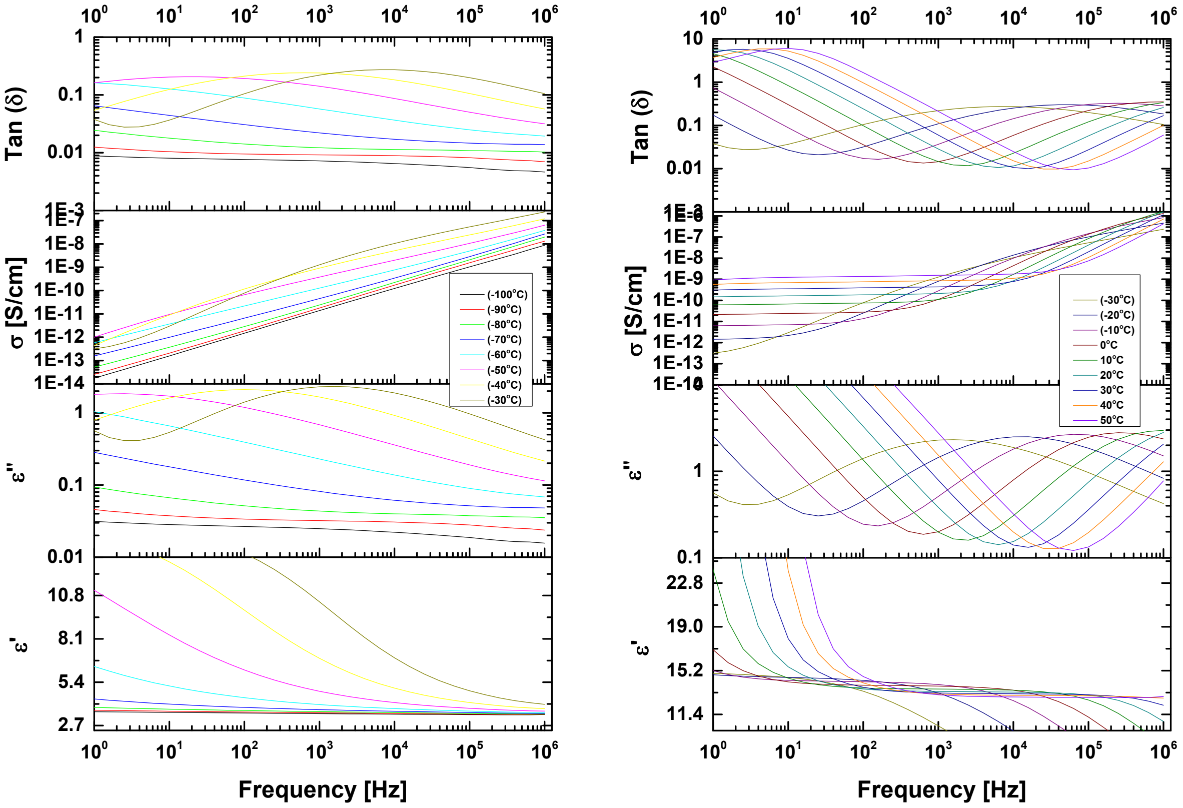

Figure S34: Dielectric properties of the ***co*-P-NA(1:2)** as function of frequency at different temperature.

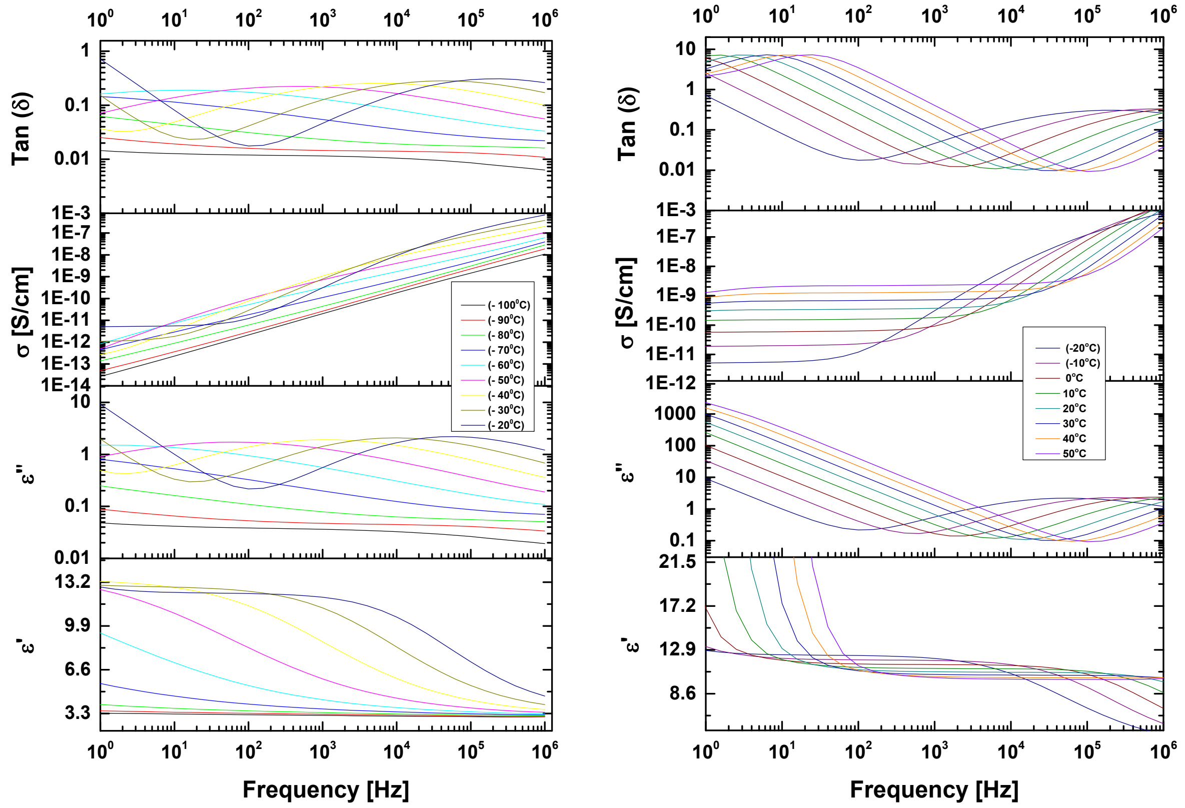

Figure S35: Dielectric properties of the ***co*-P-NA(1:1)** as function of frequency at different temperature.

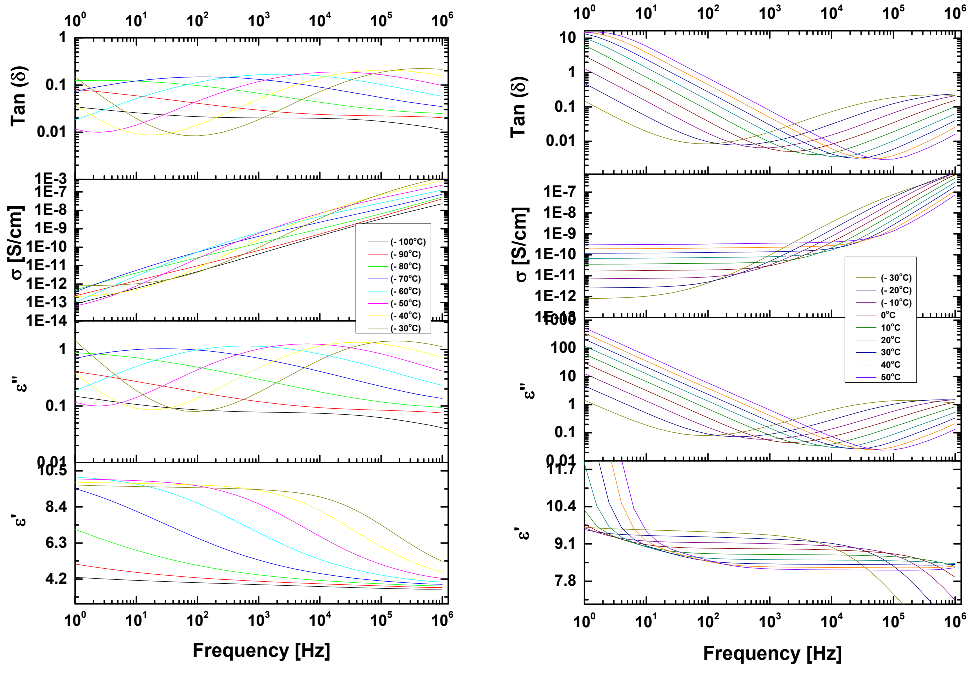

Figure S36: Dielectric properties of the ***co*-P-NA(2:1)** as function of frequency at different temperature.

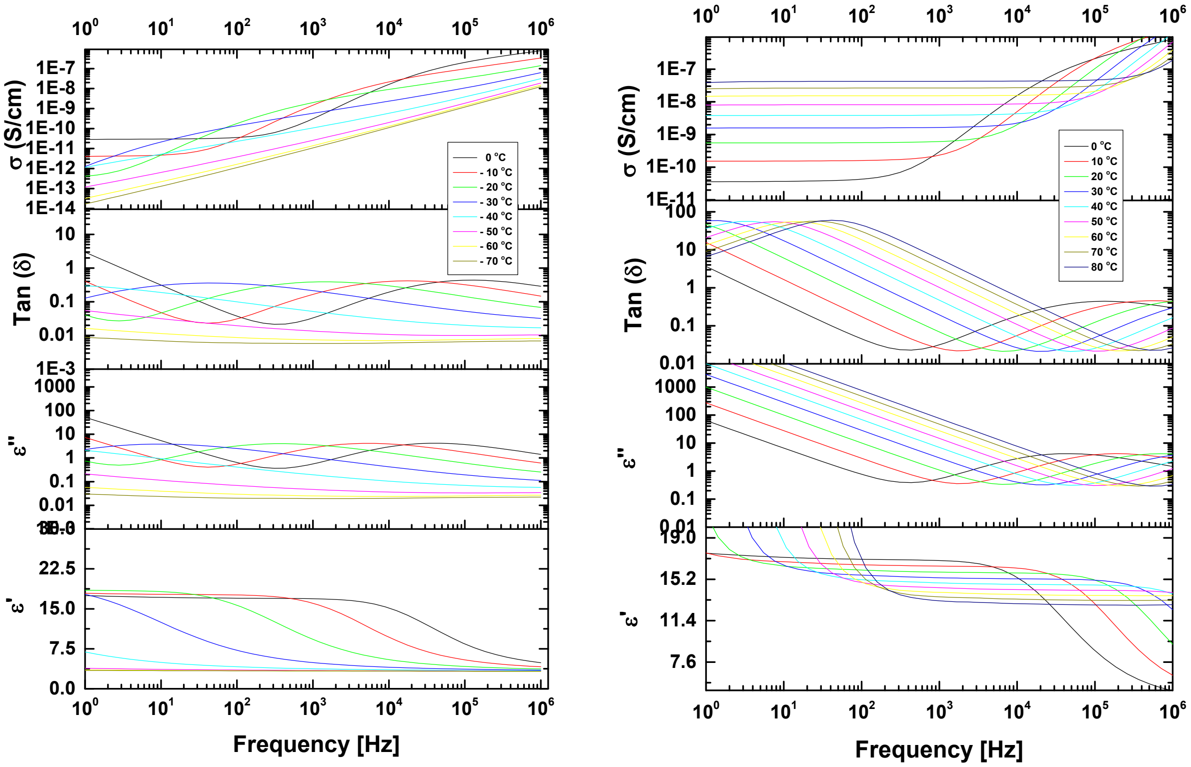

Figure S37: Dielectric properties of the **P-MNA** as function of frequency at different temperature.

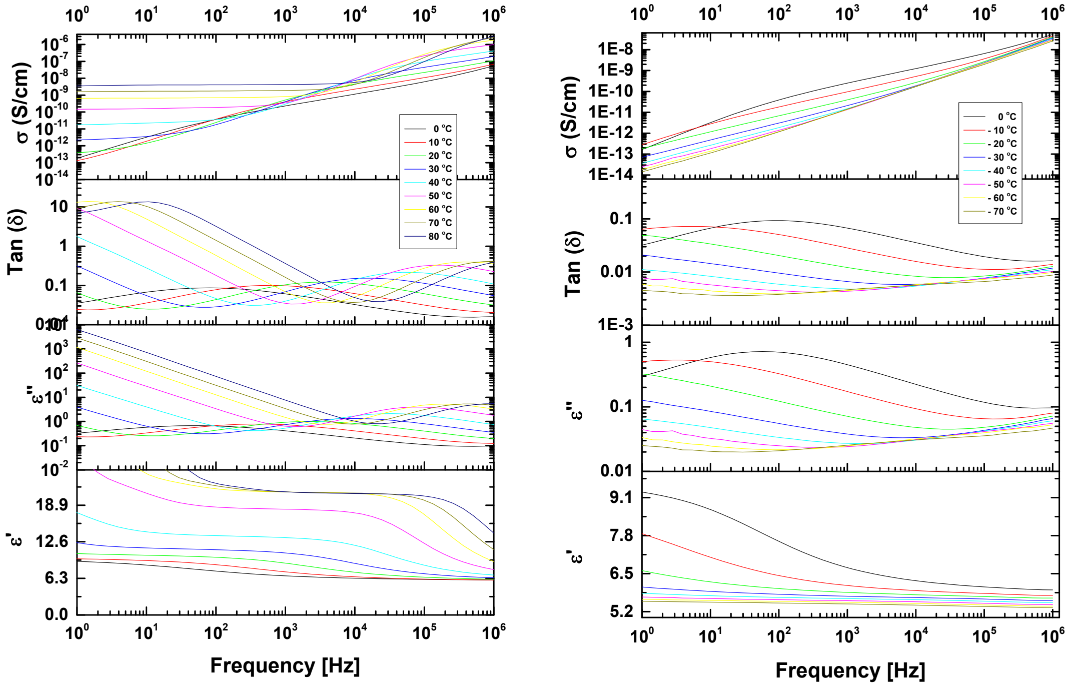

Figure S38: Dielectric properties of the **P-MDR1** as function of frequency at different temperature.

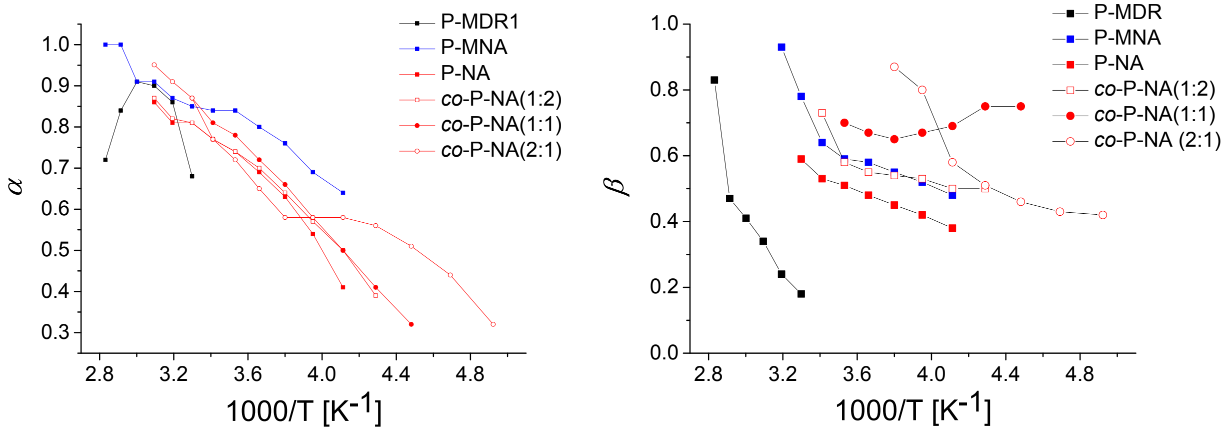

Figure S39: Characteristic exponents α (left) and β (right) of the dipolar loss peak as function of temperature.

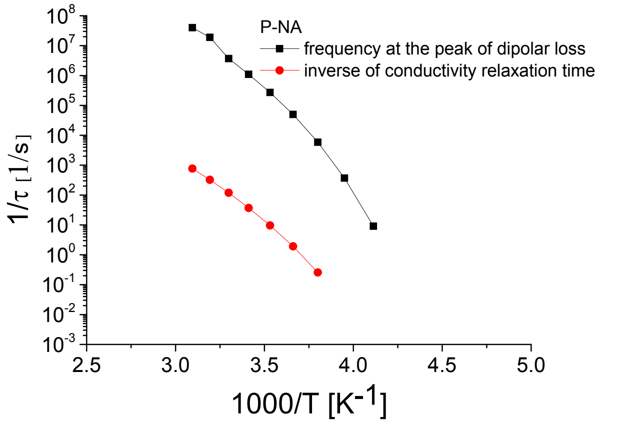

Figure 40: Frequency at the peak of dipolar loss and inverse of conductivity relaxation time as function of temperature for **P-NA**.

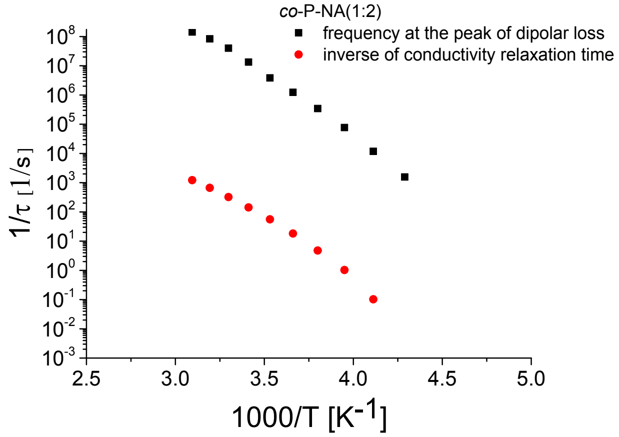

Figure 41: Frequency at the peak of dipolar loss and inverse of conductivity relaxation time as function of temperature for ***co*-P-NA(1:2)**.

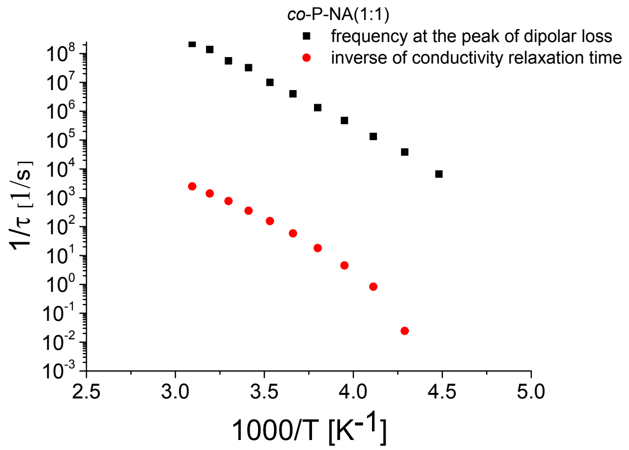

Figure 42: Frequency at the peak of dipolar loss and inverse of conductivity relaxation time as function of temperature for ***co*-P-NA(1:1)**.

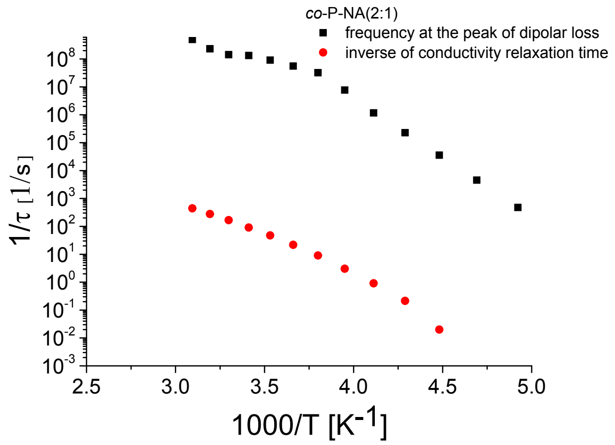

Figure 43: Frequency at the peak of dipolar loss and inverse of conductivity relaxation time as function of temperature for ***co*-P-NA(2:1)**.

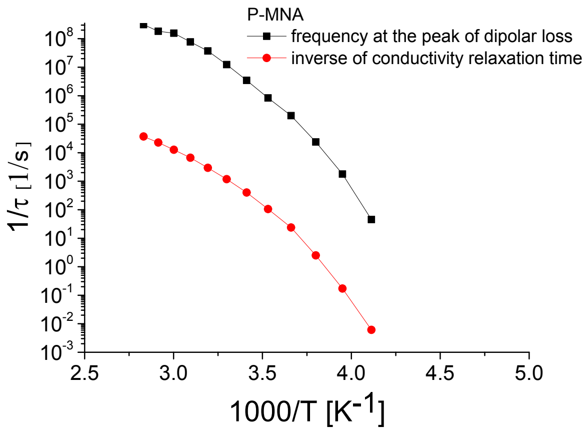

Figure 44: Frequency at the peak of dipolar loss and inverse of conductivity relaxation time as function of temperature for **P-MNA**.

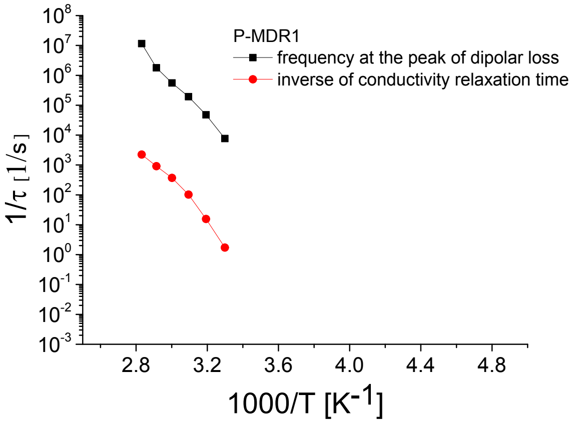

Figure 45: Frequency at the peak of dipolar loss and inverse of conductivity relaxation time as function of temperature for **P-**MDR1.

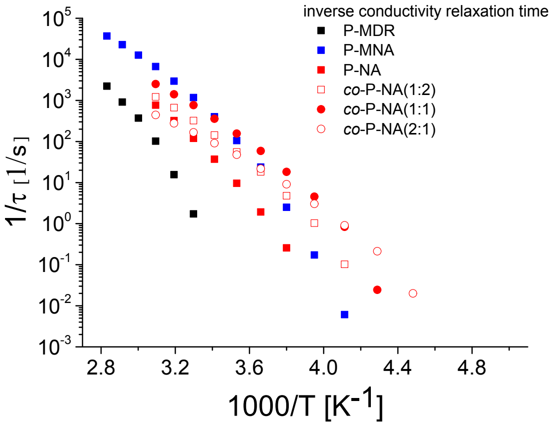

Figure 46: Inverse of conductivity relaxation time as function of temperature for all polymers.

Table 1S. Bond distances (Å) and angles (°) for **3** and **9**.

Compound **3**.

Si1-O1	1.620(2)	Si4-C7	1.833(4)
Si1-O4	1.615(3)	O5-N2	1.234(4)
Si1-C1	1.838(4)	O6-N2	1.227(4)
Si1-C8	1.843(3)	N1-C10	1.457(4)
Si2-O1	1.609(2)	N1-C11	1.457(4)
Si2-O2	1.609(3)	N1-C12	1.363(4)
Si2-C2	1.831(4)	N2-C15	1.439(4)
Si2-C3	1.837(5)	C8-C9	1.527(5)
Si3-O2	1.607(3)	C9-C10	1.516(4)
Si3-O3	1.606(3)	C12-C13	1.402(4)

Si3-C4	1.829(5)	C12-C17	1.407(4)
Si3-C5	1.831(4)	C13-C14	1.367(4)
Si4-O3	1.609(3)	C14-C15	1.381(5)
Si4-O4	1.609(2)	C15-C16	1.383(4)
Si4-C6	1.833(4)	C16-C17	1.365(4)

O1-Si1-C1	109.90(19)	Si2-O1-Si1	153.73(16)
O1-Si1-C8	108.57(14)	Si3-O2-Si2	158.94(19)
O4-Si1-O1	108.95(14)	Si3-O3-Si4	158.8(2)
O4-Si1-C1	110.25(18)	Si4-O4-Si1	158.64(17)
O4-Si1-C8	108.59(15)	C11-N1-C10	115.4(3)
C1-Si1-C8	110.53(17)	C12-N1-C10	122.6(3)
O1-Si2-C2	107.91(17)	C12-N1-C11	120.8(3)
O1-Si2-C3	109.32(19)	O5-N2-C15	118.5(3)
O2-Si2-O1	109.98(14)	O6-N2-O5	122.5(3)
O2-Si2-C2	108.9(2)	O6-N2-C15	119.0(3)
O2-Si2-C3	109.3(2)	C9-C8-Si1	115.5(2)
C2-Si2-C3	111.3(2)	C10-C9-C8	112.0(3)
O2-Si3-C4	108.55(19)	N1-C10-C9	114.2(3)
O2-Si3-C5	109.8(2)	N1-C12-C13	121.1(3)
O3-Si3-O2	110.05(14)	N1-C12-C17	121.1(3)
O3-Si3-C4	108.1(2)	C13-C12-C17	117.9(3)
O3-Si3-C5	109.7(2)	C14-C13-C12	120.9(3)
C4-Si3-C5	110.6(2)	C13-C14-C15	120.1(3)
O3-Si4-O4	109.59(13)	C14-C15-N2	120.1(3)
O3-Si4-C6	109.88(19)	C14-C15-C16	120.3(3)
O3-Si4-C7	107.71(19)	C16-C15-N2	119.5(3)
O4-Si4-C6	109.25(19)	C17-C16-C15	119.9(3)
O4-Si4-C7	109.30(17)	C16-C17-C12	121.0(3)
C7-Si4-C6	111.1(2)		

Compound **9**.

Si1-O1	1.612(4)	N1-C16	1.369(6)
Si1-O4	1.598(4)	N4-C25	1.436(7)
Si1-C1	1.848(8)	C8-C9	1.467(8)
Si1-C8	1.847(6)	C9-C10	1.545(8)
Si2-O1	1.620(4)	C9-C11	1.519(7)
Si2-C2	1.818(8)	C12-C13	1.482(8)
Si2-C3	1.827(8)	C14-C15	1.510(9)
Si2-O2	1.626(6)	C16-C17	1.399(6)
Si3-O2	1.618(6)	C16-C21	1.397(7)
Si3-O3	1.628(5)	N2-N3	1.239(12)
Si3-C4	1.837(9)	N2-C19	1.405(9)
Si3-C5	1.833(10)	N3-C22	1.446(10)
Si4-O4	1.620(4)	C17-C18	1.382(7)
Si4-C6	1.835(9)	C18-C19	1.395(7)

Si4-C7	1.815(7)	C19-C20	1.390(6)
Si4-O3	1.626(5)	C20-C21	1.387(7)
O5-C11	1.195(6)	C23-C24	1.39
O6-C11	1.341(7)	C23-C22	1.39
O6-C12	1.461(6)	C24-C25	1.39
O7-N4	1.213(6)	C25-C26	1.39
O8-N4	1.224(6)	C26-C27	1.39
N1-C13	1.482(6)	C27-C22	1.39
N1-C14	1.441(7)		

O1-Si1-C1	109.3(3)	C8-C9-C10	114.3(5)
O1-Si1-C8	110.6(2)	C8-C9-C11	113.0(5)
O4-Si1-O1	108.8(2)	C11-C9-C10	108.2(5)
O4-Si1-C1	109.0(3)	O5-C11-O6	123.4(5)
O4-Si1-C8	111.2(3)	O5-C11-C9	126.6(5)
C8-Si1-C1	107.9(3)	O6-C11-C9	109.9(5)
O1-Si2-C2	110.0(3)	O6-C12-C13	111.0(5)
O1-Si2-C3	108.7(3)	N1-C13-C12	108.8(5)
O1-Si2-O2	109.0(4)	N1-C14-C15	113.7(5)
C2-Si2-C3	110.5(5)	N1-C16-C17	117.1(6)
O2-Si2-C2	102.4(6)	N1-C16-C21	125.3(6)
O2-Si2-C3	116.1(6)	C21-C16-C17	117.3(7)
O2-Si3-O3	106.5(6)	Si3-O2-Si2	148.0(9)
O2-Si3-C4	98.6(9)	Si4-O3-Si3	149.9(8)
O2-Si3-C5	123.8(9)	N3-N2-C19	113.1(9)
O3-Si3-C4	97.8(9)	N2-N3-C22	112.9(8)
O3-Si3-C5	122.9(9)	C18-C17-C16	120.6(10)
C5-Si3-C4	99.4(12)	C17-C18-C19	122.1(10)
O4-Si4-C6	110.2(4)	C18-C19-N2	117.2(8)
O4-Si4-C7	107.6(3)	C20-C19-N2	125.8(9)
O4-Si4-O3	110.4(3)	C20-C19-C18	117.0(8)
C7-Si4-C6	110.3(5)	C21-C20-C19	121.4(10)
O3-Si4-C6	101.3(5)	C20-C21-C16	121.2(9)
O3-Si4-C7	116.9(5)	C24-C23-C22	120.0
Si1-O1-Si2	153.3(3)	C25-C24-C23	120.0
Si1-O4-Si4	150.8(3)	C24-C25-N4	123.4(6)
C11-O6-C12	116.2(4)	C24-C25-C26	120.0
C14-N1-C13	116.8(5)	C26-C25-N4	116.5(6)
C16-N1-C13	120.3(4)	C27-C26-C25	120.0
C16-N1-C14	122.6(4)	C22-C27-C26	120.0
O7-N4-O8	123.7(6)	C23-C22-N3	124.4(6)
O7-N4-C25	116.6(6)	C27-C22-N3	115.5(6)
O8-N4-C25	119.6(7)	C27-C22-C23	120.0
C9-C8-Si1	117.6(4)		

Substanz: **2**
Molekularformel: C10 H12 N2 O2 Mr = 192,22 g/mol
Bestimmungen: C H N O

M-160916

Berechnete Gewichtsanteile:

[C]	62,49%	[H]	6,29%	[N]	14,57%	[O]	16,65%

Gefundene Gewichtsanteile:

Einwaage: 1,058mg LECO TruSpec Micro

**[C]**	**62,47%**	**[H]**	**6,27%**	**[N]**	**14,74%**

Einwaage: 2,758mg LECO RO-478

[O] 16,53%

Elemental analysis of **2**.

Substanz: **3**
Molekularformel: C17 H34 N2 O6 Si4 Mr = 474.81g/mol
Bestimmungen: C H N

M-163811

Berechnete Gewichtsanteile:

[C]	43.00%	[H]	7.22%	[N]	5.90%	[O]	20.22%	[Si]	23.66%

Gefundene Gewichtsanteile:

Einwaage: 0.997mg LECO TruSpec Micro

**[C]**	**42.97%**	**[H]**	**7.18%**	**[N]**	**6.02%**

Elemental analysis of **3**.

Substanz: **5**
Molekularformel: C13 H16 N2 O4 Mr = 264.28g/mol
Bestimmungen: C H N

M-163825

Berechnete Gewichtsanteile:

[C] 59.08% [H] 6.10% [N] 10.60% [O] 24.22%

Gefundene Gewichtsanteile:

Einwaage: 1.128mg LECO TruSpec Micro
[C] 58.81% [H] 6.14% [N] 10.64%

Elemental analysis of **5**.

Substanz: **6**
Molekularformel: C20 H38 N2 O8 Si4 Mr = 546.87g/mol
Bestimmungen: C H N

M-163832

Berechnete Gewichtsanteile:

[C] 43.93% [H] 7.00% [N] 5.12% [O] 23.41% [Si] 20.54%

Gefundene Gewichtsanteile:

Einwaage: 1.253mg LECO TruSpec Micro
[C] 43.71% [H] 6.94% [N] 5.00%

Elemental analysis of **6**.

Substanz: **8**
Molekularformel: C20 H22 N4 O4 Mr = 382.42g/mol
Bestimmungen: C H N

M-163826

Berechnete Gewichtsanteile:

[C] 62.82% [H] 5.80% [N] 14.65% [O] 16.73%

Gefundene Gewichtsanteile:

Einwaage: 1.015mg LECO TruSpec Micro
[C] 62.84% [H] 5.89% [N] 14.36%

Elemental analysis of **8**.

Substanz: **9**
Molekularformel: C27 H44 N4 O8 Si4 Mr = 665.01g/mol
Bestimmungen: C H N

M-163821

Berechnete Gewichtsanteile:

[C]	48.77%	[H]	6.67%	[N]	8.42%	[O]	19.25%	[Si]	16.89%

Gefundene Gewichtsanteile:

Einwaage: 0.919mg LECO TruSpec Micro
[C] 49.03% [H] 6.72% [N] 8.44%

Elemental analysis of **9**.

Substanz: **P-NA**
Molekularformel:
Bestimmungen: C H N

M-160927

Berechnete Gewichtsanteile: ---

Gefundene Gewichtsanteile:

Einwaage: 1,539mg LECO TruSpec Micro
[C] 43,16% [H] 7,45% [N] 6,54%

Einwaage: 1,546mg LECO TruSpec Micro
[C] 43,33% [H] 7,41% [N] 6,43%

Elemental analysis of **P-NA**.

Substanz: **co-P-NA(1:2)**
Molekularformel:
Bestimmungen: C H N

M-160930
Berechnete Gewichtsanteile: ---

Gefundene Gewichtsanteile:

Einwaage: 1,557mg LECO TruSpec Micro
[C] 41,30% [H] 7,83% [N] 4,71%

Einwaage: 1,559mg LECO TruSpec Micro
[C] 41,14% [H] 7,76% [N] 4.67%

Elemental analysis of **co-P-NA(1:2)**.

Substanz: **co-P-NA(1:1)**
Molekularformel:
Bestimmungen: C H N

M-160928
Berechnete Gewichtsanteile: ---

Gefundene Gewichtsanteile:

Einwaage: 1,474mg LECO TruSpec Micro
[C] 38,98% [H] 7,80% [N] 4,05%

Einwaage: 1,519mg LECO TruSpec Micro
[C] 38,93% [H] 7,86% [N] 3,89%

Elemental analysis of **co-P-NA(1:1)**.

Substanz: **co-P-NA(2:1)**
Molekularformel:
Bestimmungen: C H N

M-160929

Berechnete Gewichtsanteile: ---

Gefundene Gewichtsanteile:

Einwaage: 1,539mg LECO TruSpec Micro

[C] 37,67% [H] 8,12% [N] 2,76%

Einwaage: 1,522mg LECO TruSpec Micro

[C] 37,48% [H] 8,19% [N] 2,93%

Elemental analysis of **co-P-NA(2:1)**.

Substanz: **P-MNA**
Molekularformel: C20 H38 N2 O8 Si4 Mr = 546.87g/mol
Bestimmungen: C H N

M-163828

Berechnete Gewichtsanteile:

[C] 43.93% [H] 7.00% [N] 5.12% [O] 23.41% [Si] 20.54%

Gefundene Gewichtsanteile:

Einwaage: 1.013mg LECO TruSpec Micro

[C] 43.81% [H] 7.07% [N] 5.20%

Elemental analysis of **P-MNA**.

Substanz: **P-MDR1**
Molekularformel: C27 H44 N4 O8 Si4 Mr = 665.01g/mol
Bestimmungen: C H N

M-163813

Berechnete Gewichtsanteile:

[C] 48.77% [H] 6.67% [N] 8.42% [O] 19.25% [Si] 16.89%

Gefundene Gewichtsanteile:

Einwaage: 0.880mg LECO TruSpec Micro

[C] 48.64% [H] 6.75% [N] 8.67%

Elemental analysis of **P-MDR1**.
